# Targeted deletion of EMMPRIN in microglia/macrophages mitigates neuronal death in intracerebral hemorrhage

**DOI:** 10.1186/s13024-025-00917-x

**Published:** 2025-12-13

**Authors:** Zhe Li, Xiangyu Zhang, Maryam Mobarakabadi, Yang Liu, Ruixue Wei, Claudia Silva, Frank Visser, Antoine Dufour, Daniel Young, Deepak Kaushik, V. Wee Yong, Mengzhou Xue

**Affiliations:** 1 Department of Cerebrovascular Diseases, The Second Affiliated Hospital of Zhengzhou University, Zhengzhou, Henan China; 2 Academy of Medical Science, Zhengzhou University, Zhengzhou, Henan China; 3 Hotchkiss Brain Institute and Department of Clinical Neurosciences, University of Calgary, Calgary, AB Canada; 4 Departments of Biochemistry and Molecular Biology, and Physiology and Pharmacology, and McCaig Institute for Bone and Joint Health, University of Calgary, Calgary, Canada; 5 Division of Biomedical Sciences, Faculty of Medicine, Memorial University of Newfoundland, St. John’s, Canada

**Keywords:** Intracerebral hemorrhage, EMMPRIN, Microglia/macrophages, Matrix metalloproteinases, Neuroprotection, Regeneration

## Abstract

**Background:**

Intracerebral hemorrhage (ICH) is a devastating subtype of stroke with high mortality and limited therapeutic options. Microglia and macrophages are rapidly recruited to the lesion site and contribute substantially to secondary brain injury. However, the key molecular mediators that drive their neurotoxic effects remain incompletely understood.

**Methods:**

We investigated the role of extracellular matrix metalloproteinase inducer (EMMPRIN, also known as CD147) in promoting microglia/macrophage-mediated neurotoxicity after ICH. EMMPRIN was selectively deleted in myeloid cells using both AAV-mediated knockdown and CX3CR1^Cre^:EMMPRIN^fl/fl^ mice. Neuronal survival and functional outcomes were assessed using histological, molecular, and behavioral analyses.

**Results:**

Targeted deletion of EMMPRIN in microglia/macrophages significantly reduced neuronal death and improved neurological recovery following ICH. Mechanistically, EMMPRIN-mediated neurotoxicity was associated with elevated expression of matrix metalloproteinases and enhanced activation of the p38 mitogen-activated protein kinase (MAPK) pathway, and with downstream engagement of myocyte enhancer factor 2 C (MEF2C) and B-cell lymphoma 2 (Bcl2). Notably, EMMPRIN deletion also enhanced neurogenesis and oligodendrogenesis in the perihematomal region, suggesting a potential role in promoting endogenous brain repair.

**Conclusions:**

These findings establish EMMPRIN elevation in myeloid cells as a prominent regulator of ICH pathophysiology and a promising therapeutic target to limit secondary injury and promote brain repair.

**Graphical Abstract:**

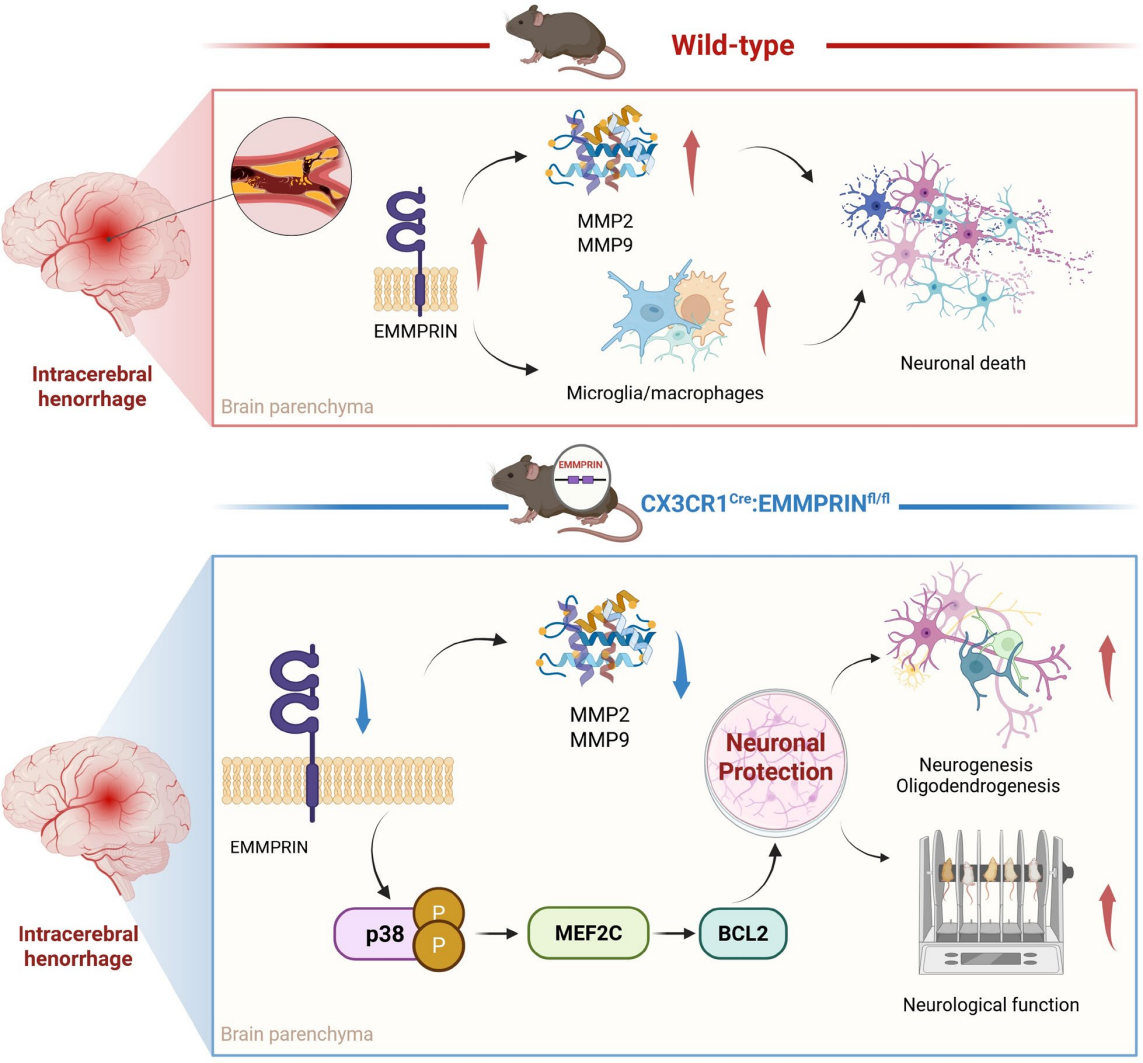

**Supplementary Information:**

The online version contains supplementary material available at 10.1186/s13024-025-00917-x.

## Background

Intracerebral hemorrhage (ICH) is one of the most devastating subtypes of stroke. It is characterized by the spontaneous rupture of cerebral blood vessels and subsequent accumulation of blood within the brain parenchyma, culminating in acute neurological dysfunction [1, 2]. Globally, the annual incidence of ICH is estimated at approximately 15–25 cases per 100,000 individuals. Despite advances in supportive care, ICH is associated with a 30-day mortality rate of 40–50%, and survivors often experience severe and long-lasting neurological deficits, underscoring its devastating clinical prognosis [3–5]. Notably, as ICH progresses, myeloid cells particularly microglia and macrophages (M/M) are rapidly activated and recruited to the perihematomal region, releasing high levels of pro-inflammatory cytokines and chemokines [6, 7]. Extensive preclinical evidence document that microglia/macrophages play a pivotal role in the pathogenesis of secondary brain injury following ICH [8, 9]. Accordingly, microglia/macrophages are a critical target for elucidating the mechanisms underlying ICH progression and for developing potential therapeutic interventions.

Matrix metalloproteinases (MMPs) are a family of zinc- and calcium-dependent endopeptidases with prominent functions in the degradation of extracellular matrix (ECM) components [10, 11]. In the context of ICH, prominent elevation of MMPs by several cell types including microglia/macrophages contributes to secondary brain injury by disrupting blood-brain barrier (BBB) integrity through the proteolytic degradation of tight junction proteins and basement membrane constituents [12]. Activation of microglia/macrophages and recruitment of peripheral immune cells further enhance MMP synthesis and release, which in turn amplifies protease and chemokine activation, promotes hematoma expansion, and induces neuronal death, thereby perpetuating a vicious cycle of damage [10, 13]. Thus, given their central role in BBB disruption and inflammation, MMPs are regarded as promising therapeutic targets to mitigate secondary injury post-ICH. However, to date, selective inhibitors targeting specific MMP isoforms in ICH remain to be identified.

Extracellular matrix metalloproteinase inducer (EMMPRIN), also known as CD147, is a highly glycosylated transmembrane protein that functions as a potent upstream regulator of MMPs [14]. EMMPRIN has been shown to induce the expression and secretion of multiple MMP enzymes, including MMP-1, MMP-2, MMP-3, and MMP-9 [15–17]. In the CNS, EMMPRIN is highly expressed during embryonic and early postnatal stages, supporting brain development and synaptogenesis [18]. Mice with constitutive EMMPRIN deletion exhibit reproductive failure, cognitive impairments, and neurobehavioral deficits, highlighting its essential role in neural development and function [19, 20]. However, its robust elevation in adult CNS injuries is linked to pathology, and previous studies have identified EMMPRIN as a key regulator in traumatic brain injury [21], Alzheimer’s disease [22], and multiple sclerosis [23]. In preclinical models of stroke, EMMPRIN is implicated as a pathogenic factor in both subarachnoid hemorrhage (SAH) [24] and transient middle cerebral artery occlusion (tMCAO) [25]. Clinical studies further demonstrate that elevated serum EMMPRIN levels correlate with poor stroke prognosis [26]. However, the cellular expression, specific role and underlying mechanisms of EMMPRIN in ICH remain largely undefined.

In the current study, we aimed to investigate the impact of targeted EMMPRIN deletion on brain injury and neuronal death following ICH. We utilized adeno-associated virus (AAV)-mediated EMMPRIN knockdown, and transgenic mice with inducible and conditional deletion of EMMPRIN from CX3CR1^+^ myeloid cells. With these mice after ICH injury, we describe a critical role for EMMPRIN elevation in modulating MMP expression, neuronal death and regenerative processes. Unbiased proteomics implicate the p38 MAPK/MEF2C signaling pathway downstream of EMMPRIN pathophysiology in myeloid cells. These findings provide new insights into the pathological function of EMMPRIN in hemorrhagic stroke and highlight its potential as a therapeutic target.

## Methods

### Ethics statement

All animals were housed in individually ventilated cages under controlled temperature and humidity conditions, with a 12-hour light-dark cycle. Food and water were provided ad libitum. All experiments were conducted with ethical approval from the Animal Care Committee at the University of Calgary (approval number: AC21-0073) in compliance with regulations set by the Canadian Council on Animal Care. All efforts were made to minimize animal suffering and the number of animals used.

### Experimental animals

A total of 42 male C57BL/6J mice (SPF grade, 20–22 g) were used to establish a collagenase-induced ICH model and to assess EMMPRIN expression. Mice were randomly assigned to seven time points post-ICH: 4 h, 8 h, 12 h, 1 day, 2 days, 3 days, and 7 days (*n* = 6), and brain tissues were harvested accordingly. For AAV-mediated knockdown experiments, 20 SPF-grade male C57BL/6J mice (25–30 g) were utilized. Two C57BL/6J mice died following AAV administration, yielding 18 animals that completed the experimental protocol. These mice were randomly divided into four groups: AAV control (day 3 post-ICH, *n* = 5), AAV knockdown (day 3 post-ICH, *n* = 5), AAV control (day 7 post-ICH, *n* = 3), and AAV knockdown (day 7 post-ICH, *n* = 5). All mice underwent behavioral tests to evaluate neurological function. CX3CR1^CreER^:Ai9 mice were euthanized at day 7 post-ICH for later analysis.

EMMPRIN^fl/fl^ mice were generated by the Yong laboratory (Cumming School of Medicine, University of Calgary) through the insertion of flox sequences flanking the 5’ region of exon 1 and the 3’ region of exon 8 of the Basigin gene. These mice have been previously described [27]. CX3CR1^CreER^ (JAX 021160) mice from Jackson Laboratory were bred in the single mouse barrier unit at the University of Calgary to produce tamoxifen-inducible CX3CR1^CreER^:EMMPRIN^fl/fl^ (EMMP-miKO) mice. The EMMPRIN^fl/fl^ and CX3CR1^CreER^ mouse lines were maintained as homozygous colonies. Both EMMPRIN^fl/fl^ and EMMP-miKO mice demonstrated 100% viability and showed no overt developmental abnormalities or health concerns. Tamoxifen (75 mg/kg; T5648, Sigma) was dissolved in corn oil (C8267, Sigma) and administered intraperitoneally once daily for five consecutive days. The ICH surgery was performed five days after the final tamoxifen injection. A total of 54 EMMP-miKO mice (8–10 weeks old males and females) were included in the ICH experiments. All mice underwent behavioral tests to evaluate neurological function. The sample size for both the control (Ctrl, without tamoxifen) group and the EMMP-miKO group (induced by tamoxifen) was *n* = 15 at day 3 post-ICH and *n* = 12 at day 7 post-ICH. Additionally, 12 EMMP-miKO mice were used for proteomic profiling. These mice were randomly divided into three experimental groups, as described in detail in the proteomics methods section.

For MEF2C knockdown, EMMP-miKO mice and their littermate controls were administered AAV vectors encoding either a non-targeting control shRNA (AAV-control) or an shRNA against MEF2C (AAV-shMEF2C). Mice were treated with tamoxifen (Tam+) or vehicle (Tam−) as described above, resulting in four groups: Tam + AAV-control (EMMPRIN conditional knockout, MEF2C intact; *n* = 6), Tam + AAV-shMEF2C (EMMPRIN conditional knockout, MEF2C knockdown; *n* = 6), Tam − AAV-control (non-conditional control, MEF2C intact; *n* = 6) and Tam − AAV-shMEF2C (non-conditional control, MEF2C knockdown; *n* = 5). All mice in these groups subsequently underwent ICH induction.

### Generation of ICH in mice

The procedure for collagenase-induced ICH in mice, approved by the Animal Care Committee of the University of Calgary and described in prior studies [28], was performed as follows. Mice were anesthetized via intraperitoneal injection of ketamine (100 mg/kg) and xylazine (10 mg/kg), and fur was removed from the surgical site using hair clippers. Ophthalmic gel was applied to both eyes to maintain moisture throughout surgery and recovery. A heat lamp positioned over the stereotaxic frame provided thermal support during the procedure. Each mouse was secured in a stereotaxic frame with modified ear bars equipped with blunt rubber ends to minimize discomfort. The surgical site was cleaned with betadine, and a skin incision was made to expose the skull. A cranial burr hole (0.5 mm) was drilled above the right striatum, located using the coordinates 2.0 mm lateral and 0.8 mm anterior to the bregma. A Hamilton syringe, loaded with 0.05 U of collagenase type VII in 0.5 µL saline, was lowered through the burr hole to a depth of 3.5 mm beneath the skull, targeting the striatum. The collagenase was injected over 5 min using a UMP3 UltraMicroPump, and the needle was left in place for an additional 5 min to minimize reflux. The needle was then carefully withdrawn, and the surgical site was sutured and disinfected. After surgery, animals were placed in a warm incubator chamber (approximately 32 °C) until fully recovered, then returned to their home cages with free access to food and water. Mice were monitored daily for post-operative health. Sham-operated animals received the same surgical procedure but were injected with 0.5 µL of saline instead of collagenase.

### Plasmid construction

The plasmid backbone pAAV-U6-GFAP-GFP-KASH-pA [29] was digested with AgeI and XbaI to excise the GFAP promoter and replaced with the CD68 monocyte promoter amplified by PCR from pAAV CD68-hM4D(Gi)-mCherry (a gift from Bryan Roth (Addgene plasmid # 75033; http://n2t.net/addgene:75033; RRID: Addgene_75033) and cloning with the NEBuilder hifi DNA assembly cloning kit (New England Biolabs). Likewise, the GFAP promoter of AAV-GFAP-SaCas9-P2A-HAFLAGHA-KASH-pA [29] was excised by digestion with XbaI and AgeI and replaced with the CD68 promoter sequence to drive SaCas9 expression. Potential single-guide RNAs (sgRNAs) with the SaCas9 PAM sequence (NNGRR) targeting the mouse EMMPRIN or MEF2C genes, respectively, were designed using the Broad Institute CRISPick web tool [30, 31]. Complementary oligonucleotides with appropriate overhang sequences and 5′ phosphorylation modifications 5′-P- ACC G GTACTTCGTATGCAGGTCGGG-3′ and 5′-P- AACCCCGACCTGCATACGAAGTACC-3′ (EMMPRIN) or 5’P-ACCG CTCCGCCCATCAGACCGCCTA and 5’P-AAC TAGGCGGTCTGATGGGCGGAG C (MEF2C) were annealed and subcloned into BspQI-digested pAAV-U6-GFAP-GFP-KASH-pA. For the nontarget control, sgRNA targeting a lacZ sequence was used [32]. All plasmid constructs were verified by restriction enzyme mapping and Sanger DNA sequencing.

### AAV production

AAV viral vectors containing the PHP.eB capsid were generated using the methods of Challis et al. [33]. PHP.eB capsid has been engineered to efficiently transduce the central nervous system [34]. Briefly, 293FT cells (Thermo Fisher Scientific) were grown to about 90% confluence in hyperflasks (Corning) and cotransfected with 129 µg pHELPER (Agilent), 238 µg rep-cap plasmid encoding PHP.eB pUCmini-iCAP-PHP.eB was a gift from Viviana Gradinaru (Addgene plasmid # 103005 ; http://n2t.net/addgene:103005 ; RRID: Addgene_103005)), and 64.6 µg of transfer plasmid using the PEIpro transfection reagent (Polyplus). AAVs were precipitated from medium harvested after 3 days and 5 days using 40% PEG/2.5 M NaCl in buffer containing 500 mM NaCl, 40 mM Tris base, and 10 mM MgCl2. The lysate was incubated with 100 U/mL salt-active nuclease (Arcticzymes) at 37 °C for 1 h and then centrifuged at 2,000 g for 15 min. AAV was purified from the resulting lysate using an iodixanol step gradient containing 15%, 25%, 40%, and 60% iodixanol in OptiSeal tubes (Beckman Coulter) followed by centrifugation at 350,000 g using a Type 70 Ti ultracentrifuge rotor (Beckman Coulter). After centrifugation, the AAVs were harvested from the 40% layer using a 10 mL syringe and 16-gauge needle, diluted in 1× PBS containing 0.001% Pluronic F68 (Gibco), and filtered using a 0.2 μm syringe filter. The AAVs were concentrated and buffer-exchanged by 5 rounds of centrifugation using Amicon Ultra-15 100-kDa molecular weight cutoff centrifugal filter units (MilliporeSigma). The titer was determined using the qPCR Adeno-Associated Virus Titration kit (Applied Biological Materials), and the purity was verified by SDS-PAGE and total protein staining using InstantBlue reagent (Expedeon).

### Flow cytometry of brain tissue

At 3 days after ICH, mice were deeply anesthetized, transcardially perfused with ice-cold PBS, and the hemorrhagic hemisphere was rapidly dissected. Brain tissue was mechanically dissociated in ice-cold HBSS by gentle trituration, then passed through a 70-µm cell strainer, and mononuclear cells were enriched by removing myelin in a three-step 90%/37%/30% Percoll gradient. After washing and counting, cells were resuspended in FACS buffer (PBS, 2% FBS) and incubated with a fixable viability dye (Fixable Viability Stain 620, BD Horizon) followed by Fc receptor blocking (anti-CD16/32, BD Biosciences; 1:100, 20 min at 4 °C). Cells were then stained with fluorochrome-conjugated antibodies against CD45, CD11b, Ly6G, Ly6C, CD11c, CD3, F4/80, B220, MHC-II, CD86 and CD206; the full list of clones, fluorophores and dilutions is provided in Supplementary Table S1. Finally, samples were washed and analyzed by Attune NxT Flow Cytometer (ThermoFisher) equipped with Attune NxT software (v3.1.2) and FlowJo software.

### Brain tissue Preparation

At selected time points post-ICH, mice were anesthetized with an overdose of ketamine and xylazine, followed by cardiac perfusion with 15 mL of PBS and 15 mL of 4% paraformaldehyde (PFA). Whole brains were carefully dissected from the skulls, fixed overnight in 4% PFA, and transferred to a 30% sucrose solution for dehydration. After 2–3 days in the sucrose solution, the brains were trimmed to approximately 2–3 mm on either side of the lesion center and embedded in FSC 22 cryosection medium (Leica). Coronal sections of brain tissue (20 μm thick) were prepared using a cryostat (ThermoFisher Scientific), mounted on Superfrost Plus microscope slides (VWR), and stored at -20 °C until analysis.

### Antibodies

The details of the primary and secondary antibodies used in this study are summarized in Supplemental Table S2.

### Immunofluorescence staining

Brain sections were equilibrated to room temperature (RT) for 30 min prior to staining. Tissue permeabilization was performed using 0.2% Triton X-100 in PBS for 10 min. To minimize nonspecific binding, sections were incubated in blocking solution (PBS supplemented with 10% horse serum, 1% BSA, 0.1% cold water fish skin gelatin, 0.1% Triton X-100, and 0.05% Tween-20) for 1 h at RT. For staining of mouse tissues, Fc receptors were blocked using a purified rat anti-mouse CD16/CD32 antibody (1:100 dilution; BD Biosciences, 553142) included in the blocking buffer. Primary antibodies were diluted in antibody dilution buffer (PBS containing 1% BSA, 0.1% cold water fish skin gelatin, and 0.1% Triton X-100) and incubated with sections overnight at 4 °C. The following day, sections were washed three times in PBS with 0.2% Tween-20 (5 min per wash) and subsequently incubated with fluorophore-conjugated secondary antibodies for 1 h at RT. After three additional washes in PBS with 0.2% Tween-20, coverslips were mounted using Fluoromount-G (SouthernBiotech) and allowed to cure before imaging.

### Confocal Immunofluorescence microscopy and image analysis

Confocal immunofluorescence imaging was performed at room temperature using a Leica TCS SP8 laser-scanning confocal microscope equipped with a 25×/0.5 NA water-immersion objective. Fluorophores were excited using 405 nm, 488 nm, 552 nm, and 640 nm laser lines, with fluorescence signals detected by two low-dark-current Hamamatsu PMT detectors and two high-sensitivity hybrid detectors. Image acquisition was performed using a z-stack with unidirectional scanning, a 1 Airy unit pinhole, a 0.75× zoom, an optical section thickness of 0.57 μm, and 2048 × 2048 pixels x-y resolution. To ensure consistency, imaging parameters were standardized across all experiments. Whole-brain section overviews at various time points post-surgery were obtained using an Olympus VS120 slide scanner with a 20× objective under identical laser and exposure conditions. Image processing was conducted using OlyVIA (Olympus), with brightness and contrast adjustments applied uniformly to enhance visualization without altering original data integrity.

Confocal image z-stacks were processed and analyzed using ImageJ. Maximum-intensity projections were generated from lif files, with pseudocolor applied to individual channels or markers. All images were analyzed in an eight-bit format. Regions of interest (ROI) were delineated to encompass the perihematomal region, lesion core and contralateral hemisphere based on GFAP or Iba1 staining, while regions outside the defined ROI were excluded from analysis. Thresholding for positive signal detection was standardized across samples using values determined from negative secondary antibody controls, as well as contralateral controls. Positive signal quantification within each ROI was performed using the analyze particles function in ImageJ, with consistent brightness thresholding, particle size, and circularity parameters maintained across all experimental groups. For representative images, only brightness and contrast adjustments were uniformly applied to enhance visualization without altering data integrity. Maximum-intensity projections from each channel within a z-stack were merged and converted to RGB format using ImageJ.

### Eriochrome cyanine and neutral red staining

Eriochrome cyanine (EC) and neutral red staining were employed for histological visualization of lesion areas. Cryosections were air-dried before undergoing sequential dehydration and rehydration steps, including treatment with CitriSolv (Fisher Scientific), isopropanol, and a graded ethanol series (100%, 95%, 90%, 70%, and 50%), each for 1 min. Following rehydration, sections were rinsed in distilled water for 1 min and incubated in EC staining solution containing 10% FeCl₃ for 15 min. After a brief rinse in distilled water, sections were differentiated in 0.5% NH₄OH for 10 s, followed by another distilled water wash. Neutral red staining (1%) was applied for 2 min to enhance contrast, with a final rinse in distilled water. Sections were then subjected to a dehydration series (50%, 70%, 90%, 95%, and 100% ethanol, each for 1 min), followed by isopropanol (2 min) and CitriSolv (4 min). Coverslips were mounted using Acrytol mounting medium (Electron Microscopy Science) and allowed to dry before imaging. Brightfield images were captured using an Olympus VS120 Slide Scanner with a 20×/0.75 NA air objective. Regions of interest (ROIs) were delineated based on areas of reduced EC staining surrounded by intensified neutral red staining, primarily within the basal ganglia. Quantitative lesion area analysis was conducted using CellSens software (Olympus).

### Behavioral tests

Motor coordination and balance were evaluated using the rotarod test, while forelimb strength was assessed using the grip strength test. Testing was conducted before surgery and at 1, 3, 5, and 7 days post-surgery. To ensure acclimatization, mice were brought to the testing room 30 min prior to each session.

### Rotarod test

Mice were trained on a rotating rod for three consecutive days prior to experimental testing to ensure familiarity with the apparatus. During the test, the rotation speed was gradually increased from 4 to 40 revolutions per minute (rpm) over a maximum 300-second period. Each mouse was placed on the rod, and the latency to fall was recorded as the primary outcome measure, with longer latencies indicating better motor coordination and balance. To prevent fatigue-related effects, a maximum cutoff time of 300 s was established. Each mouse underwent three trials, with a 10-minute rest interval between trials to allow for recovery. The average latency to fall across trials was calculated for statistical analysis. The test was performed in a quiet, controlled environment to minimize external disturbances and ensure consistent conditions.

### Grip strength

Mice were allowed to use their forelimbs to grasp a crossbar, which was gently pulled away by the experimenter. The experimental apparatus (grip strength meter) automatically recorded the peak force exerted at the moment the mouse lost its grip. Each mouse underwent multiple trials, and the average force across trials was calculated for statistical analysis.

### Experimental groups and sample preparation for shotgun proteomic analysis

For this part of experiment, mice were divided into three groups (*n* = 4 per group): (1) Sham group (control): C57BL/6J mice received daily intraperitoneal injections of tamoxifen for five consecutive days prior to surgery to exclude potential off-target influence of tamoxifen. These mice underwent stereotaxic procedures identical to the ICH model but received equal volumes of sterile saline instead of collagenase. (2) ICH + EMMP-miKO group (EMMPRIN conditional knockout): CX3CR1^creER^: EMMPRIN^fl/fl^ mice were treated with tamoxifen for five consecutive days prior to surgery to induce EMMPRIN deletion specifically in microglia/macrophages. (3) ICH group: CX3CR1^creER^:EMMPRIN^fl/fl^ mice did not receive tamoxifen and underwent ICH induction to maintain EMMPRIN expression. Perihematomal brain tissue was collected on day 3 post-surgery from all mice and subjected to shotgun proteomic analysis.

### Shotgun proteomics

Total protein was extracted by homogenization in lysis buffer (2% SDS, 200mM ammonium bicarbonate, protease inhibitor tablets, 0.1 mM EDTA) and quantified using the BCA Protein Array Kit (Pierce, Rockford, IL, USA). Samples were then prepared using the filter-assisted separation of peptides (FASP) method. 100 µg of protein was precipitated by adding trichloroacetic acid (TCA) followed by an incubation on ice for 30 min. Samples were then centrifuged at 14,000 g for 15 min at 4 °C, washed 3 times in ice cold acetone, and stored at -20 °C overnight. Samples were resuspended in 8 M tris-urea solution by shaking and then denatured with the addition of 10 mM DTT at 37 °C for 30 min. 50 mM iodoacetamide was added in the dark at room temperature to complete carbamidomethyl modification of the cystines. Samples were moved to the top of a 30 kDa filter and were then centrifuged at 14,000 g for 15 min. They were then washed 3 times with 100 µl of 8 M tris-urea and 3 washes in 100 µl of 50 mM ammonium bicarbonate. The samples were trypsinized at 37 °C overnight at a ratio of 1:10 trypsin: total protein, and subsequently eluted off the filter membrane by washing with 50 mM ammonium bicarbonate.

### High performance liquid chromatography (HPLC) and mass spectrometry

Tryptic peptides were analyzed on an Orbitrap Fusion Lumos Tribrid mass spectrometer (Thermo Scientific) operated with Xcalibur (version 4.4.16.14) and coupled to a Thermo Scientific Easy-nLC (nanoflow Liquid Chromatography) 1200 system. A total mass of 2 µg tryptic peptides were loaded onto a C18 trap (75 μm x 2 cm; Acclaim PepMap 100, P/N 164946; ThermoScientific) at a flow rate of 2µl/min of solvent A (0.1% formic acid in LC-MS grade water). Peptides were eluted using a 120 min gradient from 5 to 40% (5% to 28% in 105 min followed by an increase to 40% B in 15 min) of solvent B (0.1% formic acid in 80% LC-MS grade acetonitrile) at a flow rate of 0.3 µL/min and separated on a C18 analytical column (75 μm x 50 cm; PepMap RSLC C18; P/N ES803; ThermoScientific). The mass spectrometry was operated in DIA mode using the default settings from the manufacturer. Peptides were then electrosprayed using 2.1 kV voltage into the ion transfer tube (300 °C) of the Orbitrap Lumos operating in positive mode. The Orbitrap first performed a full scan at a resolution of 120,000 FWHM to detect the precursor ion having a *m*/*z* between 380 and 985 and a + 2 to + 7 charge. The Orbitrap AGC (Auto Gain Control) and the maximum injection time were set at standard and 50 ms, respectively. The Orbitrap was operated using the top speed mode with a 3 s cycle time for precursor selection. The most intense precursor ions presenting a peptidic isotopic profile and having an intensity threshold of at least 5000 were isolated using the quadrupole and fragmented with HCD (30% collision energy) in the ion routing multipole. The fragment ions (MS^2^) were analyzed in the orbitrap with a 40 sliding windows of 16.0072 m/z starting at 379.4224. The AGC and the maximum injection time were set at 1e4 and 35 ms, respectively, for the quadrupole.

### Differential protein screening and bioinformatics analysis

Differentially expressed proteins were identified based on a fold change (FC) > 1.2 or < 0.83 and a p-value < 0.05, indicating at least a 1.2-fold upregulation or downregulation with statistical significance. These criteria were used to select proteins for further functional annotation and pathway analysis. KEGG and Gene Ontology (GO) enrichment analyses were performed using the clusterProfiler and org.Mm.eg.db R packages. For KEGG analysis, the top 20 significantly enriched pathways were selected for visualization. GO enrichment was conducted across three categories: Biological Process (BP), Cellular Component (CC), and Molecular Function (MF), with the top 10 enriched terms in each category visualized in enrichment plots. To explore potential interactions among the differentially expressed proteins, protein–protein interaction (PPI) networks were constructed using the STRING database and visualized using Cytoscape software, providing insight into the functional connectivity and interaction landscape of the proteomic alterations.

### PRIDE

The mass spectrometry proteomics data have been deposited to the ProteomeXchange Consortium via the PRIDE partner repository with the dataset identifier PXD063205.

### Statistical analyses

Data collection and organization were performed using Microsoft Excel, while statistical analyses were conducted with GraphPad Prism 10.0.3. To ensure data transparency and reproducibility, individual data points for each group are presented in statistical graphs. Data were presented as mean ± standard error of the mean (SEM). Prior to analysis, normality was assessed using the Shapiro-Wilk test. For normally distributed data, homogeneity of variance was further evaluated. If variance was homogeneous, group comparisons were performed using a two-tailed unpaired t-test. In cases of unequal variance, Welch’s correction was applied to the t-test to account for variance heterogeneity. For datasets that did not pass the normality test, intergroup comparisons were conducted using the Mann-Whitney U test. Multiple-group comparisons were performed using one-way analysis of variance (ANOVA) followed by Tukey’s post hoc test for multiple comparisons. Statistical significance was indicated using asterisks: *p < 0.05, **p < 0.01, ***p < 0.001.

## Results

### Elevation of EMMPRIN in microglia/macrophages following ICH

**Fig. 1:**
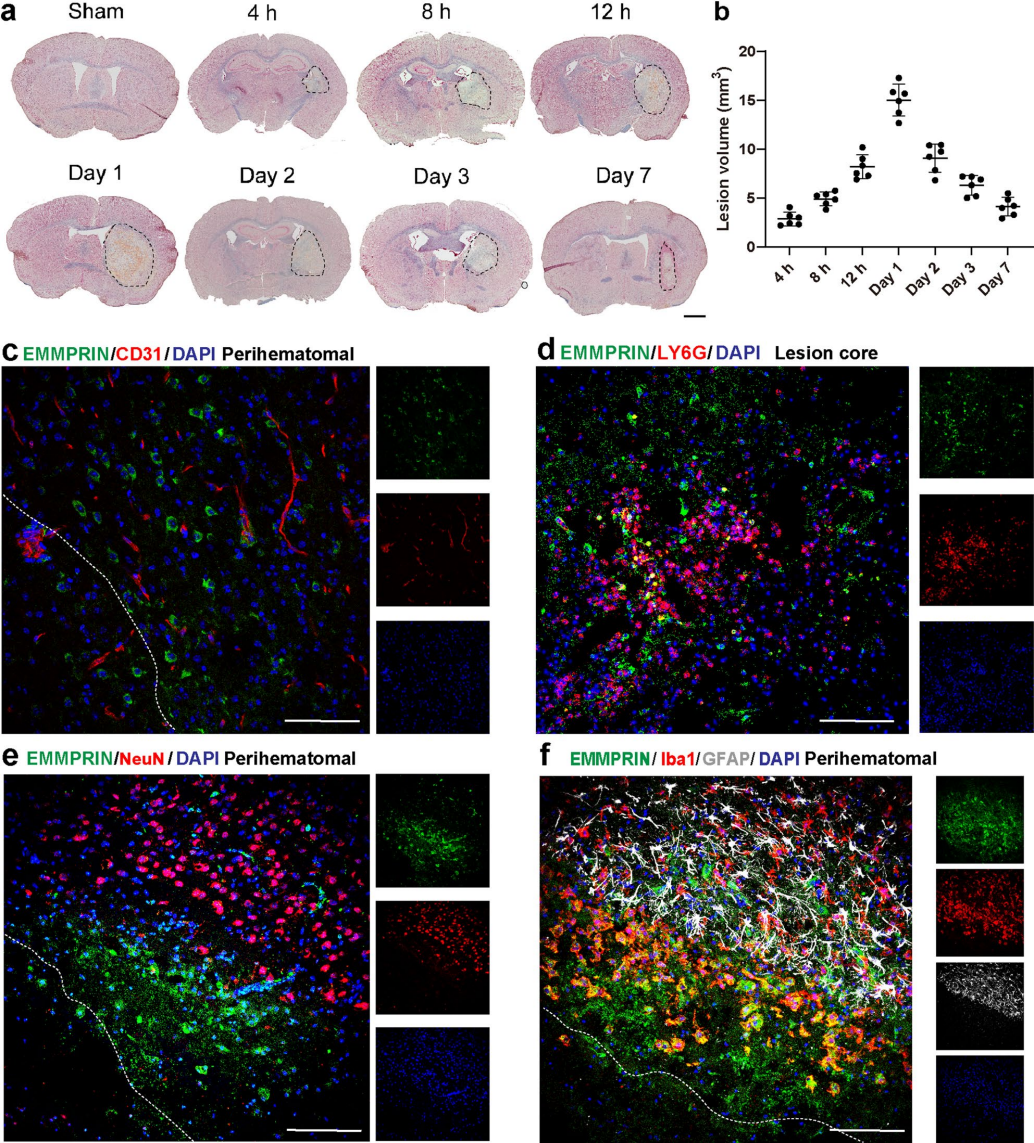
Temporal evolution of lesion volume and cellular localization of EMMPRIN following ICH. **(a)** Representative eriochrome cyanine-stained coronal brain sections from sham and ICH mice at 4–12 h, and 1–7d post-ICH. Hematoma boundaries are delineated by dashed lines. Scale bar, 1 mm. **(b)** Quantification of lesion volume across time points (*n* = 6 per group). **(c–f)** Representative confocal images of EMMPRIN (green) with endothelial cells (CD31, c), neutrophils (Ly6G, d), neurons (NeuN, e), and microglia/astrocytes (Iba1 in red, GFAP in gray, f). Images are 3 days post-ICH and at the perihematomal region except for neutrophils that are found in the lesion core. The left corner within the dotted line of the perihematomal region indicates the center of the lesion. Scale bar, 100 μm. Sample size in each group was *n* = 6 mice. Data are presented as the mean ± SEM and analyzed by one-way ANOVA-Tukey’s post hoc test; ns: not significant. Significance is indicated as *p < 0.05, **p < 0.01, ***P < 0.001

To assess the temporal evolution of brain injury post-ICH, eriochrome cyanine and neutral red stainings were performed at defined intervals. Hematoma formation was evident at 4 h post-ICH, expanded over time, and peaked at day 1. A gradual reduction in lesion volume was observed thereafter, with significant shrinkage by day 7 associated with resorption of hematoma (Fig. 1a, b). To delineate the cellular localization of EMMPRIN, we performed immunofluorescence co-staining with various cell-type markers (Fig. 1c-f). At day 3 post-ICH, EMMPRIN did not co-localize with endothelial cells (CD31), neurons (NeuN), or astrocytes (GFAP) in the perihematomal region. Ly6G⁺ neutrophils were abundant in the lesion core, and EMMPRIN signal did not overlap with them. In contrast, strong co-localization of EMMPRIN with Iba1⁺ microglia/macrophages were observed in the perihematomal region (Fig. 1f).

**Fig. 2:**
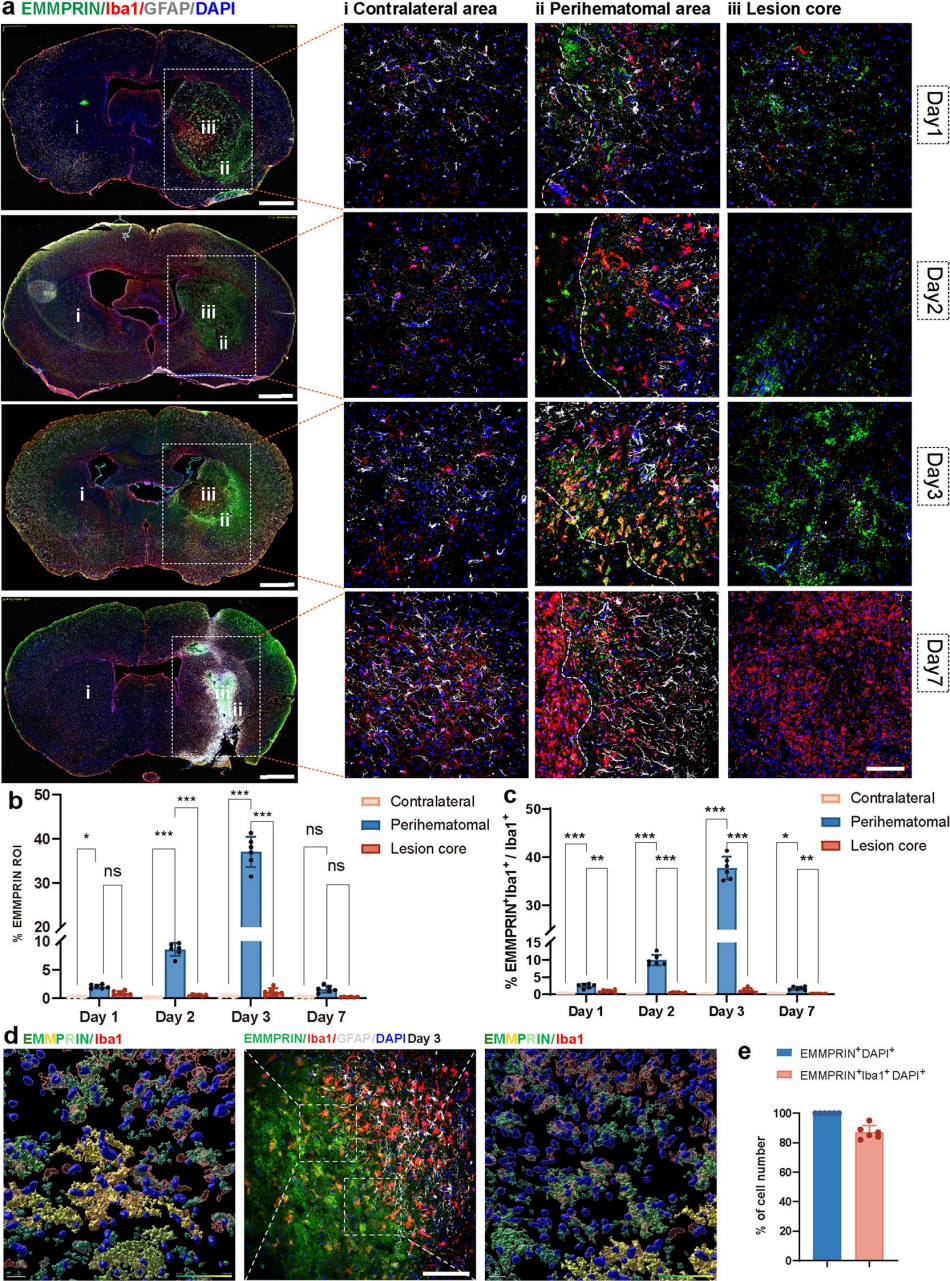
EMMPRIN level increased and co-localized with microglia/macrophages following ICH. **(a)** Representative images of EMMPRIN/Iba1 and GFAP staining at days 1, 2, 3, and 7 post-ICH, with areas depicted as contralateral unaffected hemisphere (i), perihematomal region (ii) and lesion core (iii) enlarged in the right panels. The left corner within the dotted line of the perihematomal region indicates the center of the lesion. Scale bar for left panel is 1 mm, while that for the right panel is 100 μm. **(b**,** c)** Quantification of % EMMPRIN in the region of interest (ROI) (**b**), and the proportion of Iba1^+^ microglia/macrophages expressing EMMPRIN (**c**) at corresponding time points post-ICH in the perihematomal region, lesion core and contralateral hemisphere. **(d)** Representative image of a three-dimensional (3D) reconstruction of EMMPRIN (green) co-localized with Iba1⁺ (red) at day 3 post-ICH. The central panel shows the perihematomal region in 2D immunofluorescence, while the left and right panels display high-magnification 3D Imaris reconstruction of labels. Scale bar: 100 μm (center) and 20 μm (left and right). **(e)** Quantification showing the percentage of EMMPRIN⁺ cells co-expressing Iba1⁺ within the EMMPRIN⁺DAPI⁺ cell population. Sample size in each group was *n* = 6 mice. Data are presented as the mean ± SEM and analyzed by one-way ANOVA-Tukey’s post hoc test; ns: not significant. Significance is indicated as *p < 0.05, **p < 0.01, ***P < 0.001

To assess EMMPRIN expression in microglia/macrophages, immunofluorescence microscopy was performed at days 1, 2, 3, and 7 post-ICH, focusing on the perihematomal region, lesion core and contralateral hemisphere (Fig. 2a). Quantification showed low EMMPRIN levels across all regions on day 1. Expression significantly increased in the perihematomal region on day 2 (*p* < 0.001), peaked on day 3 (*p* < 0.001), and returned to baseline by day 7 (Fig. 2b). Similarly, the percentage of Iba1⁺ cells co-expressing EMMPRIN rose significantly in the perihematomal region on day 2 (*p* < 0.001), peaked on day 3 (*p* < 0.001), and declined by day 7 (Fig. 2c), indicating a temporally regulated and region-specific upregulation of EMMPRIN in microglia/macrophages during acute ICH.

To further validate the cellular specificity of EMMPRIN expression, we performed Imaris-based 3D reconstruction of the perihematomal region at day 3 post-ICH. This improved visualization over conventional 2D imaging and revealed clear spatial co-localization of EMMPRIN with Iba1⁺ microglia/macrophages (Fig. 2d). Quantification (Fig. 2e) showed that ~ 90% of EMMPRIN⁺DAPI⁺ cells were Iba1⁺, confirming EMMPRIN’s predominant localization in microglia/macrophages.

### EMMPRIN expression correlates with MMP expression and neuronal death

**Fig. 3:**
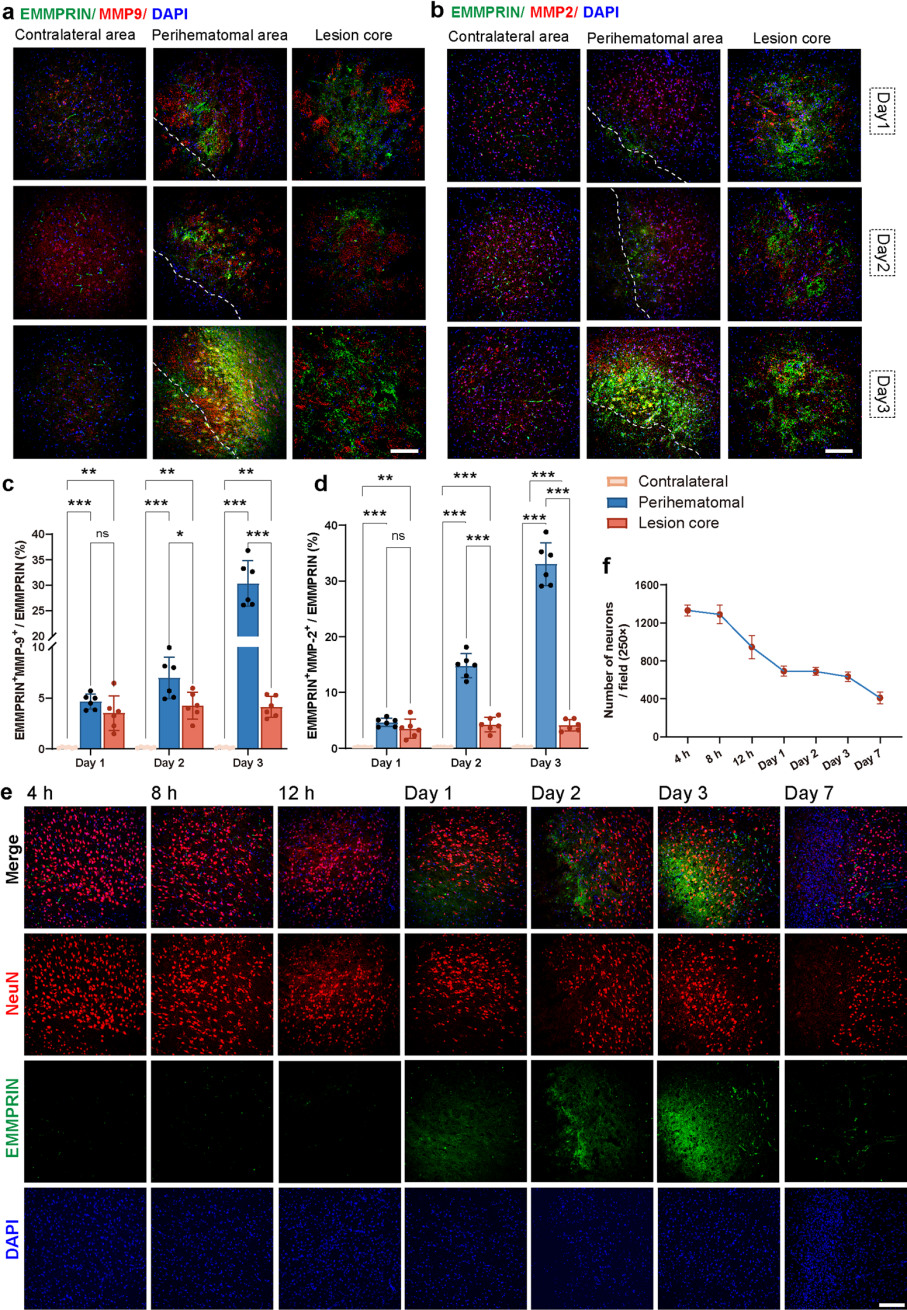
EMMPRIN expression correlated with MMPs expression and neuronal death. **(a-b)** Representative confocal images of EMMPRIN (green) co-localized with MMP-9 (red, a) and MMP-2 (red, b) in the contralateral hemisphere, perihematomal region and lesion core at days 1, 2, and 3 post-ICH. The left corner within the dotted line of the perihematomal region indicates the center of the lesion. Scale bar, 100 μm. **(c-d)** Quantification showing the percentage of EMMPRIN co-localized with MMP-9 (c) and MMP-2 (d) in different brain regions and time points post-ICH. **(e)** Representative confocal images of temporal dynamics of EMMPRIN (green) and NeuN (red, neuronal marker) at 4 h, 8 h, 12 h, 1 day, 2 days, 3 days, and 7 days post-ICH. Scale bar, 100 μm. Sample size in each group was *n* = 6 mice. Data are presented as the mean ± SEM and analyzed by one-way ANOVA-Tukey’s post hoc test; ns: not significant. Significance is indicated as *p < 0.05, **p < 0.01, ***P < 0.001. **(f)** Quantification of NeuN^+^ cell numbers (mean ± SEM, n of 6) at corresponding time points post-ICH, reflecting the temporal progression of neuronal death

Immunofluorescence analyses revealed increased co-expression of EMMPRIN with MMP-9 and MMP-2 in the perihematomal region beginning on day 1, and more obvious at days 2 and 3 post-ICH (Fig. 3a, b). Quantification showed low but significantly different (comparing perihematomal or lesion core values to that in the contralateral hemisphere) elevation and co-localization of EMMPRIN with MMP-2 or -9 on day 1, which increased further on day 2 and 3 particularly in the perihematomal region (Fig. 3c, d). These findings indicate that EMMPRIN may facilitate MMP-9 and MMP-2 induction, contributing to extracellular matrix degradation and BBB disruption post- ICH.

Next, we evaluated neuronal survival in ICH pathology by counting NeuN⁺ cells in the perihematomal region from 4 h to 7 d (Fig. 3e, f). A drop in neuronal count was apparent after 12 h of ICH and was marked at day 1 (Fig. 3f) when EMMPRIN expression was beginning to be detectable by immunofluorescence microscopy (Fig. 3e).

### Targeted reduction of EMMPRIN by AAV improves ICH outcomes and promote phenotype change of microglia/macrophages following ICH

**Fig. 4:**
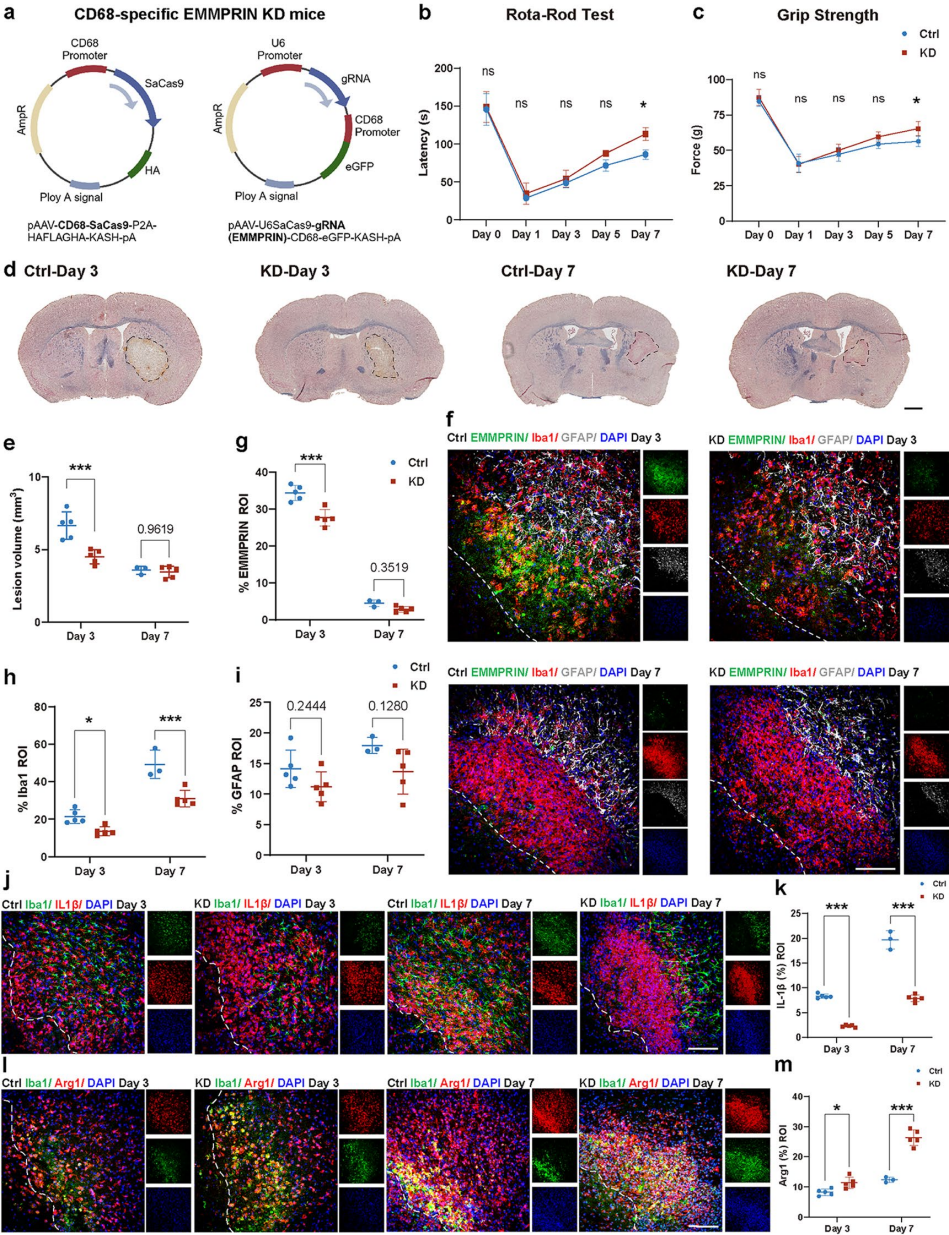
Targeted reduction of EMMPRIN by AAV ameliorates ICH outcome and promote phenotype change of microglia/macrophages. **(a)** Schematic showing the genome of AAVs used in this study. AAVs were injected retro-orbitally at 3 × 10^11^ vg/virus per mouse 2 weeks before ICH for targeted reduction of EMMPRIN (KD) or non-target guide RNA (Ctrl). **(b**,** c)** Graphs comparing the latency of rota-rod test (b) or forelimb force of grip strength test (c) between Ctrl and KD groups. **(d)** Representative images of EC staining at day 3 and day 7 post-ICH. Scale bar = 1 mm. **(e)** Bar graph comparing the day 3 and day 7 lesion volume calculated from EC staining among Ctrl and KD groups. **(f)** Representative confocal images of EMMPRIN/Iba1/GFAP/DAPI staining in the perihematomal region in Ctrl and KD groups at day 3 and day 7 post-ICH. The left corner within the dotted line of the perihematomal region indicates the center of the lesion. Scale bar, 100 μm. **(g-i)** Quantification showing the percentage of EMMPRIN (g), Iba1 (h), GFAP (i) in lesion ROI between Ctrl and KD mice. **(j**,** l)** Representative confocal images of Iba1/IL1β/DAPI (j) and Iba1/Arg1/DAPI (l) staining in perihaematomal area in Ctrl and KD groups at day 3 and day 7 post-ICH. The left corner within the dotted line of the perihematomal region indicates the center of the lesion. Scale bar = 100 μm. **(k**,** m)** Bar graphs comparing the percentage of IL1β (k) and Arg1 (m) in lesion ROI between Ctrl and KD groups at day 3 and day 7 post-ICH. Except for the control group at day 7 post-ICH, which was *n* = 3 mice, sample size in all other groups was *n* = 5 mice each. Data are presented as the mean ± SEM and analyzed by one-way ANOVA-Tukey’s post hoc test; ns: not significant. Significance is indicated as *p < 0.05, ***P < 0.001

To assess the impact of elevation of EMMPRIN post-ICH, an AAV-coupled CRISPR/Cas9 approach was used to target EMMPRIN expression in microglia/macrophages given the predominant expression of EMMPRIN in Iba1^+^ cells noted above. We generated 2 different AAVs: (1) recombinant PHPeB AAV vector packaging a specific gRNA for targeted disruption of EMMPRIN (U6-EMMPRINgRNA-CD68-eGFP), and (2) recombinant PHPeB AAV vector encoding Cas9 under the control of a CD68 promoter (CD68-SaCas9-P2A-HAFLAGHA) (Fig. 4a). AAVs (3 × 10^11^ viral genomes (vg) per virus) were co-delivered retro-orbitally to mice 2 weeks before ICH induction. Control mice were injected with AAV encoding non-target guide RNA (U6-lacZgRNA-CD68-eGFP). Following ICH, while all mice had a drop in rota-rod latency and grip strength, which then gradually recovered, the AAV-EMMPRIN knockdown (KD) mice had better recovery at day 7 of ICH (Fig. 4b, c).

Histological analyses were evaluated after 3 or 7 days of ICH in the AAV EMMPRIN KD or control groups. There was a markedly decreased lesion volume and %EMMPRIN in ROI at day 3 post-ICH in the EMMPRIN KD compared to control group (Fig. 4d-f). However, this was not maintained at day 7 of ICH, likely because the hematomal lesion volume had resolved prominently in all animals, and because EMMPRIN elevation at day 7 was returning to control levels.

The reduction of EMMPRIN in ICH by AAV was accompanied by a decrease in the density of microglia/macrophages while GFAP^+^ astrocytes were unaffected, at both day 3 and 7 of ICH (Fig. 4h, i). In addition, the KD group exhibited a significant decrease in the expression of pro-inflammatory IL-1β (Fig. 4j, k), while arginase-1 (Arg1) normally associated with regulatory microglia/macrophages was elevated (Fig. 4l, m). These results suggest a shift of microglia/macrophages toward a more regulatory or reparative phenotype.

### AAV targeted reduction of EMMPRIN attenuates MMPs level and neuronal death following ICH

**Fig. 5:**
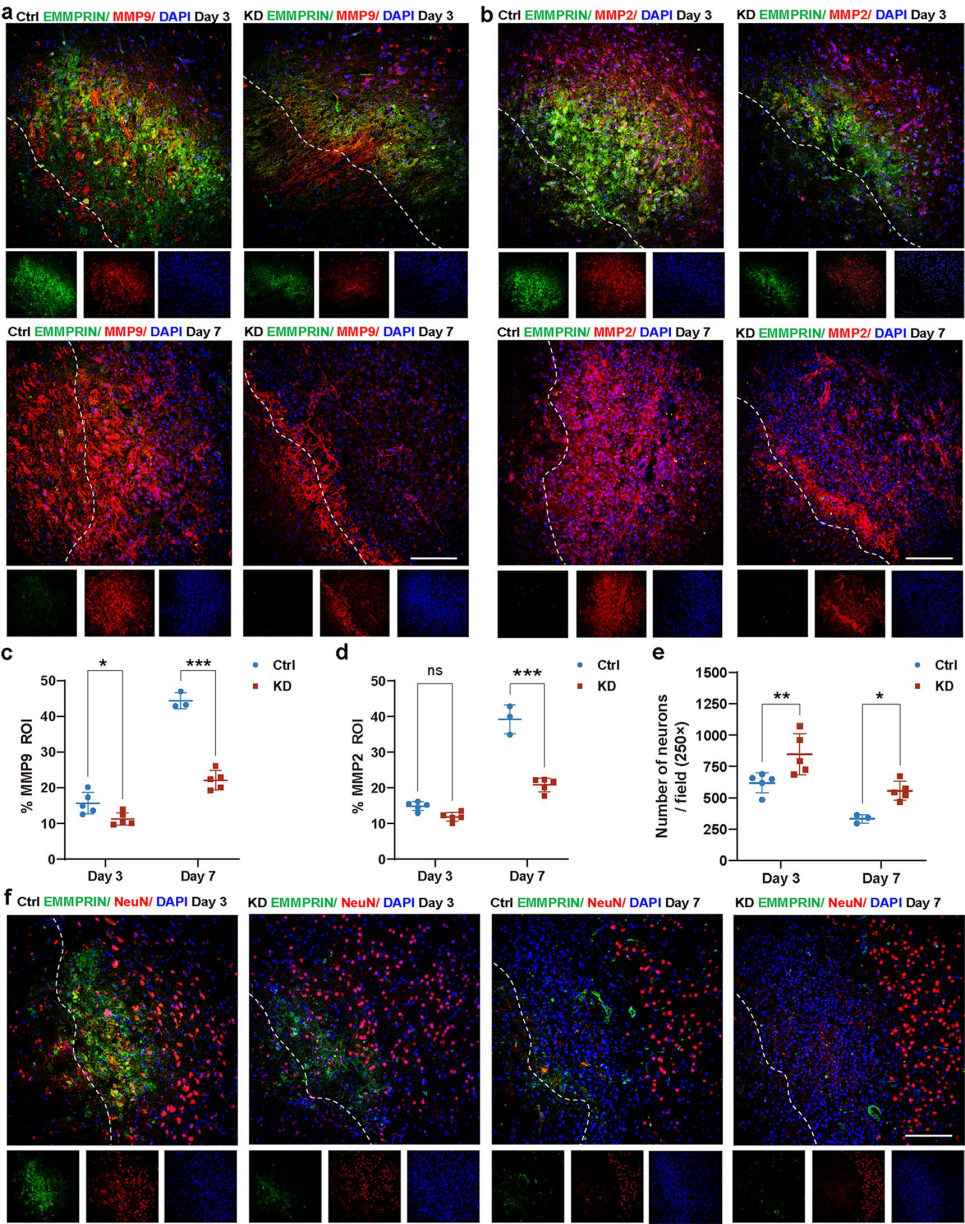
Targeted reduction of EMMPRIN attenuates MMPs level and neuronal death following ICH. **(a**,** b)** Representative confocal images of EMMPRIN/MMP9/DAPI (a) and EMMPRIN/MMP2/DAPI (b) staining in the perihematomal region in Ctrl and AAV-KD groups at day 3 and day 7 post-ICH. The left corner within the dotted line of the perihematomal region indicates the center of the lesion. Scale bar, 100 μm. **(c**,** d)** Quantification of the percentage of MMP-9 (c), MMP-2 (d) in lesion ROI between Ctrl and KD mice. **(e)** Bar graph compares the density of neurons between Ctrl and KD mice at day 3 and day 7 post-ICH. Except for the control group at day 7 post-ICH, which was *n* = 3 mice, sample size in all other groups was *n* = 5 mice each. Data are presented as the mean ± SEM and analyzed by one-way ANOVA-Tukey’s post hoc test; ns: not significant. Significance is indicated as *p < 0.05, **p < 0.01, ***P < 0.001. **(f)** Representative confocal images of EMMPRIN/NeuN/DAPI staining in the perihematomal region in Ctrl and KD groups at day 3 and 7 post-ICH. The left corner within the dotted line of the perihematomal region indicates the center of the lesion. Scale bar, 100 μm

We analyzed the expression of MMP-9 and MMP-2 in AAV-EMMPRIN KD mice (Fig. 5a, b). Here, the KD group exhibited a significant reduction in MMP-9 levels at both day 3 (*p* < 0.05) and day 7 (*p* < 0.001) post-ICH (Fig. 5c). In contrast, MMP-2 levels showed a significant decrease only at day 7 post-ICH (*p* < 0.001, Fig. 5d). Importantly, the KD group had reduced neuronal dropout compared to the control AAV group at day 3 (*p* < 0.01) and day 7 (*p* < 0.05) post-ICH (Fig. 5e, f).

### Conditional deletion of myeloid EMMPRIN in floxed mice promotes functional recovery following ICH, associated with reduced neuronal death

**Fig. 6:**
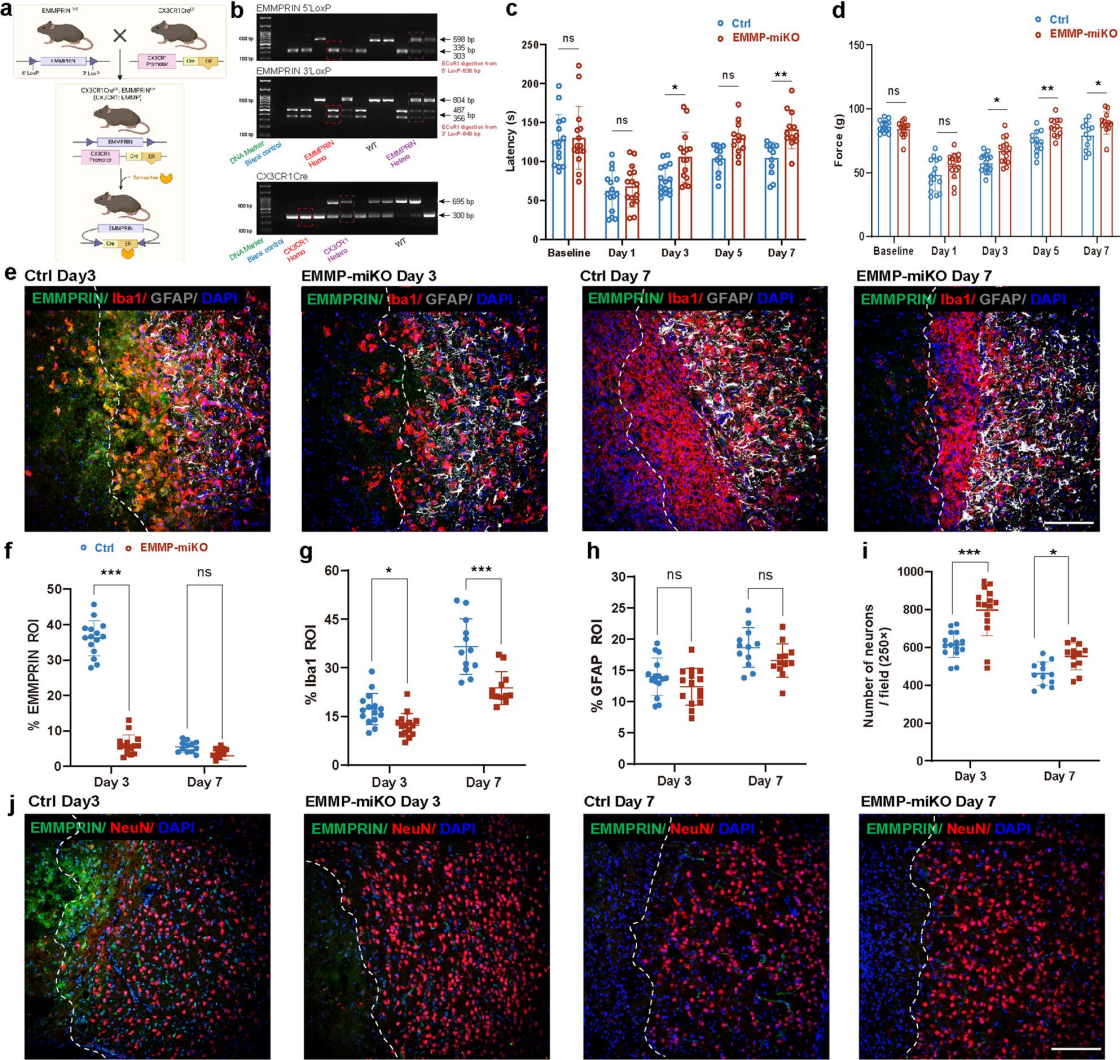
EMMPRIN deletion in microglia/macrophages alleviates neuronal death and promotes functional recovery following ICH. **(a)** Mating scheme for generating CX3CR1^CreER^:EMMPRIN ^fl/fl^ (EMMP-miKO) mice. **(b)** Representative PCR results of genotype identification. **(c**,** d)** Bar graphs comparing the latency of rota-rod test (c) or forelimb force of grip strength test (d) between Ctrl and EMMP-miKO mice at different time points before and post-ICH, each circle is a different mouse and compiled across 3 separate experiments. **(e)** Representative confocal images of EMMPRIN/Iba1/GFAP/DAPI staining in the perihematomal region in Ctrl and EMMP-miKO groups at day 3 and 7 post-ICH. The left corner within the dotted line of the perihematomal region indicates the center of the lesion. Scale bar, 100 μm. **(f-h)** Quantification showing the percentage of EMMPRIN (f), Iba1 (g), GFAP (h) in lesion ROI between Ctrl and EMMP-miKO mice. **(i)** Bar graph compares the density of neurons between Ctrl and EMMP-miKO mice at day 3 and 7 post-ICH. Sample size in the Ctrl group and the EMMP-miKO group was *n* = 15 at day 3 post-ICH; *n* = 12 at day 7 post-ICH. Data are presented as the mean ± SEM and analyzed by one-way ANOVA-Tukey’s post hoc test; ns: not significant. Significance is indicated as *p < 0.05, **p < 0.01, ***P < 0.001. **(j)** Representative confocal images of EMMPRIN/NeuN/DAPI staining in the perihematomal region in Ctrl and EMMP-miKO groups at day 3 and day 7 post-ICH. Scale bar, 100 μm

To further evaluate the role of EMMPRIN in post-ICH recovery, we used EMMPRIN^fl/fl^ mice generated recently by us for another study [27] and crossed them to CX3CR1^CreER^ mice (Fig. 6a, b). CX3CR1 is predominantly in microglia/macrophages [35], and EMMPRIN would be deleted from them after tamoxifen administration to generate mice referred henceforth as EMMP-miKO mice. In these mice, compared to control mice (CX3CR1^Cre^:EMMPRIN^fl/fl^ without tamoxifen), functional recovery assessed by rota-rod and grip strength tests were improved post-ICH (Fig. 6c, d).

To confirm the efficiency of EMMPRIN deletion, we assessed its expression in the perihematomal region at day 3 and 7 post-ICH using immunofluorescence staining (Fig. 6e). Quantitative analysis revealed a significant reduction in EMMPRIN expression in EMMP-miKO mice compared to control (Fig. 6f). At day 3 post-ICH, a period of peak EMMPRIN detection after ICH as noted above, EMMPRIN expression in the EMMP-miKO group decreased to ~ 8% in the perihematomal ROI, compared to ~ 40% in the control group (*p* < 0.001). This reduction in EMMPRIN expression was accompanied by a decrease in the Iba1^+^ representation of microglia/macrophages (Fig. 6g). Since EMMPRIN expression is minimal at day 7 post-ICH, no significant difference in EMMPRIN levels was observed between the EMMP-miKO group and the control group at this time point. Nonetheless, despite the negligible expression of EMMPRIN at this stage, the level of activated microglia/macrophages was approximately 10% lower in the EMMP-miKO group compared to the control group (*p* < 0.001), reflecting the sustained effects of microglial EMMPRIN knockout. In contrast, astrocyte activation, as indicated by GFAP staining, showed no significant differences between the groups at both day 3 and 7 post-ICH (Fig. 6h).

Since neuronal death contributes greatly to neurologic deficits, we used immunofluorescence microscopy to detect neuronal survival in the perihematomal region post-ICH (Fig. 6i, j). Compared to controls, the EMMP-miKO group had more surviving NeuN^+^ neurons at both day 3 (*p* < 0.001) and day 7 (*p* < 0.05) post-ICH (Fig. 6i).

**Fig. 7:**
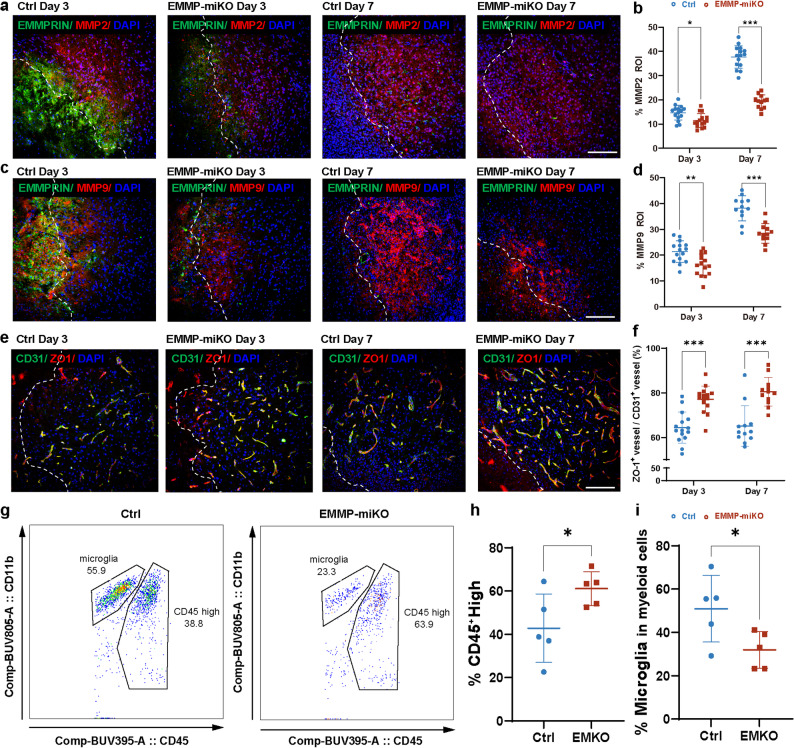
Microglia/macrophage EMMPRIN deletion attenuates MMP upregulation, preserves blood–brain barrier integrity and alters microglia composition after ICH. **(a**,** c)** Representative confocal images of EMMPRIN/MMP-2/DAPI (a) and EMMPRIN/MMP-9/DAPI (c) staining in the perihematomal region in Ctrl and EMMP-miKO groups at day 3 and 7 post-ICH. The left corner within the dotted line of the perihematomal region indicates the center of the lesion. Scale bar, 100 μm. **(b**,** d)** Quantification of the percentage of MMP-2 (b) or MMP-9 (d) in lesion ROI between Ctrl and EMMP-miKO groups. **(e)** Representative confocal images of CD31/ZO-1/DAPI staining in the perihematomal region in Ctrl and EMMP-miKO mice at day 3 and day 7 post-ICH. The left corner within the dotted line of the perihematomal region indicates the center of the lesion. Scale bar, 100 μm. **(f)** Quantification of the proportion of ZO-1⁺/CD31⁺ vessels within the CD31⁺ vascular profiles in Ctrl and EMMP-miKO groups at day 3 and 7 post-ICH. Sample size in the Ctrl group and the EMMP-miKO group was *n* = 15 on day 3 post-ICH; *n* = 12 on day 7 post-ICH. **(g)** Representative flow cytometry plots of brain leukocytes 3 days after ICH, showing CD45^int^CD11b^+^ microglia and CD45^hi^CD11b^+^ infiltrating myeloid cells in Ctrl and EMMP-miKO mice. The full gating strategy is shown in supplementary Fig. S1. **(h)** Quantification of CD45^hi^ cells as a percentage of total CD45^+^ brain leukocytes; *n* = 5 of each group. **(i)** Quantification of the proportion of microglia within the CD45⁺CD11b⁺ myeloid compartment; *n* = 5 of each group. Data are presented as the mean ± SEM and analyzed by one-way ANOVA-Tukey’s post hoc test; ns: not significant. Significance is indicated as *p < 0.05, **p < 0.01, ***P < 0.001

We examined the level of metalloproteinases and found that the EMMP-miKO group displayed a significantly lower representation of MMP-2 (Fig. 7a, b) and MMP-9 (Fig. 7c, d) immunoreactivity at both day 3 (*p* < 0.01) and day 7 (*p* < 0.001) post-ICH. These results affirm a clear lowering effect of EMMPRIN deletion on MMPs expression. In keeping with reduced MMP burden, immunostaining for the endothelial marker CD31 and the tight junction protein ZO-1 showed better preservation of vascular integrity in EMMP-miKO mice. At both day 3 and day 7 post-ICH, the proportion of ZO-1⁺/CD31⁺ vessels among total CD31⁺ vascular profiles in the perihematomal region was significantly higher in EMMP-miKO mice than in controls (Fig. 7e, f), indicating less disruption of endothelial tight junctions after EMMPRIN deletion.

To complement the histological evidence and to characterize microglial responses more precisely, we profiled brain leukocytes by flow cytometry at day 3 post-ICH. A detailed gating strategy for isolating microglia and other immune subsets is shown in Supplementary Fig. S1a, and the antibody panel is listed in Supplementary Table S1. In this dataset, frequencies of most lymphoid and myeloid subsets did not differ significantly between genotypes (Supplementary Fig. S1b-f), suggesting that the overall leukocyte repertoire was not grossly altered by EMMPRIN deletion. However, within the myeloid compartment we detected a shift in composition. EMMP-miKO mice showed a higher proportion of CD45^hi^CD11b⁺ infiltrating myeloid cells among total CD45⁺ brain leukocytes compared with controls (*p* < 0.05; Fig. 7h), whereas the fraction of CD45^int^CD11b⁺ microglia within the CD45⁺CD11b⁺ myeloid gate was reduced (*p* < 0.05; Fig. 7i). Representative plots are shown in Fig. 7g. Thus, microglia-specific EMMPRIN deletion not only dampens MMP upregulation and preserves BBB structure, but also reshapes the balance between resident microglia and infiltrating myeloid cells in the injured brain.

### EMMPRIN may exert neuroprotective effects via the p38 MAPK/MEF2C signaling pathway

**Fig. 8:**
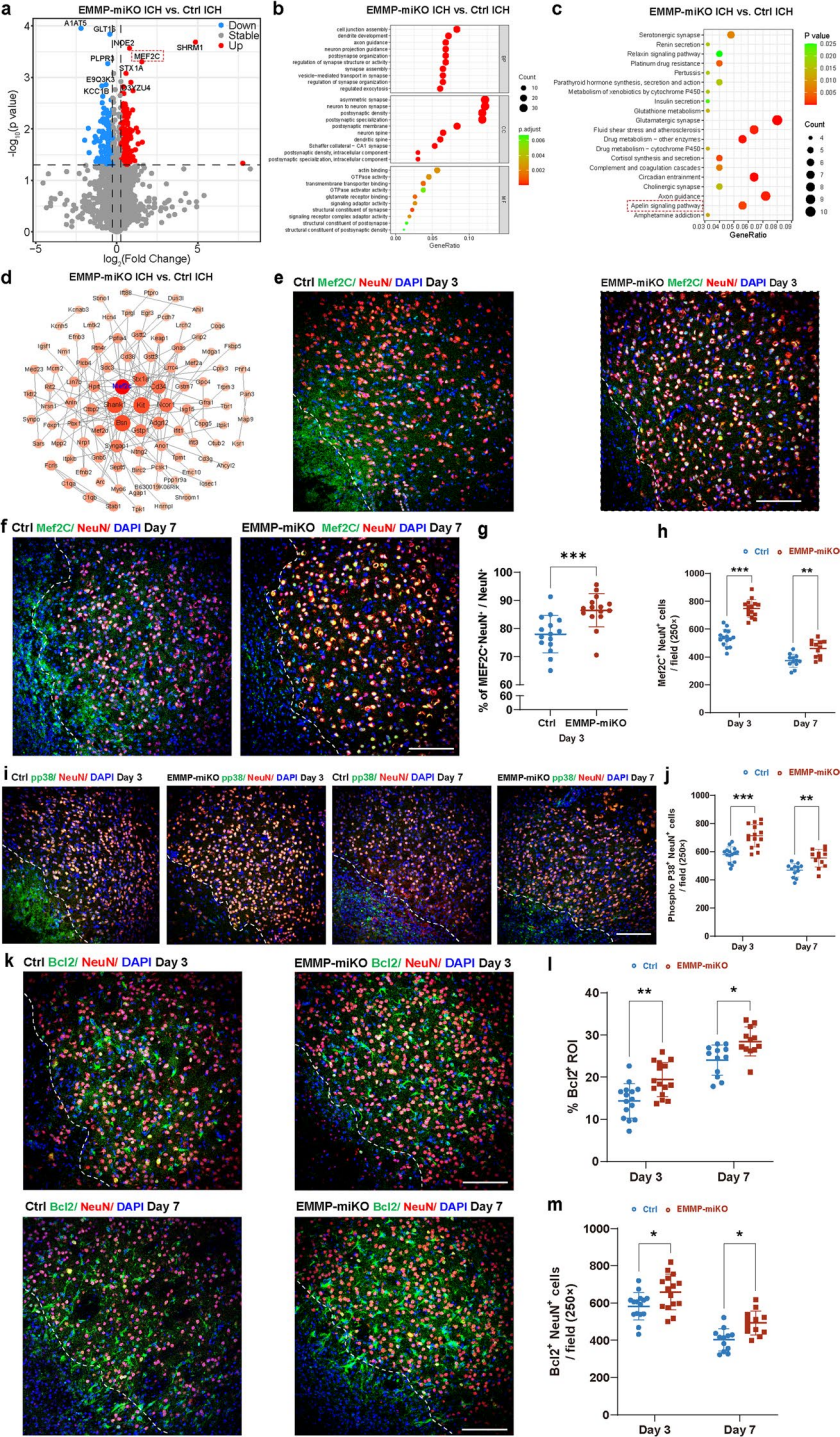
Microglial EMMPRIN deletion enhances MEF2C expression, and may exert neuroprotective effects via the p38 MAPK/MEF2C signaling pathway following ICH. **(a)** Volcano plot showing differentially expressed proteins between EMMP-miKO and ICH groups. X-axis: log₂ fold change; Y-axis: log₁₀ (p-value). Red: upregulated proteins; blue: downregulated proteins; gray: non-significant unchaged proteins. **(b)** GO enrichment analysis. X-axis: gene ratio; Y-axis: enriched GO terms. Dot size indicates gene count; color scale (green to red) reflects adjusted p-value. **(c)** KEGG pathway analysis. X-axis: gene ratio; Y-axis: enriched pathways. Dot size indicates gene count; color scale reflects p-value. **(d)** Protein–protein interaction (PPI) network. Node size indicates degree; color intensity reflects significance; edges represent predicted interactions. **(e-f)** Representative confocal images of MEF2C (green) co-expression with NeuN (red) in Ctrl and EMMP-miKO groups at day 3 and day 7 post-ICH. The left corner within the dotted line of the perihematomal region indicates the center of the lesion. Scale bar, 100 μm. **(g)** Quantification of the proportion of MEF2C⁺NeuN⁺ cells among neurons in Ctrl and EMMP-miKO groups at day 3 post-ICH. **(h)** Quantification of MEF2C⁺NeuN⁺ cell density in Ctrl and EMMP-miKO groups at day 3 and day 7 post-ICH. **(i)** Representative confocal images of phosphorylated p38 (pp38, green) co-localized with NeuN (red) in Ctrl and EMMP-miKO groups at day 3 and day 7 post-ICH. The left corner within the dotted line of the perihematomal region indicates the center of the lesion. Scale bar, 100 μm. **(j)** Quantification of pp38⁺NeuN⁺ cell density in Ctrl and EMMP-miKO groups at day 3 and day 7 post-ICH. **(k)** Representative confocal images of Bcl2 (green) co-expression with NeuN (red) in Ctrl and EMMP-miKO groups at day 3 and day 7 post-ICH. The left corner within the dotted line of the perihematomal region indicates the center of the lesion. Scale bar, 100 μm. **(l)** Quantification of the percentage of Bcl2⁺ in lesion ROI between Ctrl and EMMP-miKO groups. **(m)** Quantification of Bcl2⁺NeuN⁺ neuronal cell density in Ctrl and EMMP-miKO groups at day 3 and day 7 post-ICH. Sample size in the Ctrl group and the EMMP-miKO group was *n* = 15 on day 3 post-ICH; *n* = 12 on day 7 post-ICH. Data are presented as the mean ± SEM and analyzed by one-way ANOVA-Tukey’s post hoc test; ns: not significant. Significance is indicated as *p < 0.05, **p < 0.01, ***P < 0.001

To elucidate the mechanisms by which EMMPRIN deletion reduces ICH injury, we performed shotgun proteomics of brain tissue from EMMP-miKO and control mice at 2 days post-ICH. Volcano plot analysis revealed significant upregulation of several proteins in EMMP-miKO mice, one of which was MEF2C (log₂ fold change > 1.5; Fig. 8a). GO enrichment analysis indicated that differentially expressed proteins were associated with neuronal processes such as synapse development and postsynaptic density (Fig. 8b). KEGG analysis indicated enrichment in neuroregenerative pathways, including glutamatergic synapse, axon guidance, and Apelin signaling (Fig. 8c). PPI network analysis identified the transcription factor MEF2C as a highly connected hub gene, interacting with multiple proteins involved in neuronal development and synaptic function (Fig. 8d), suggesting its key role in EMMPRIN-related neuroprotective signaling.

We assessed the neuronal expression of MEF2C via immunofluorescence. At both days 3 and 7 post-ICH, EMMP-miKO mice had significantly increased co-localization of MEF2C with NeuN⁺ neurons compared to controls. Consistently, when normalized to total neurons, the proportion of MEF2C-expressing neurons was markedly increased to 87% in EMMP-miKO mice (Fig. 8e-h).

Next, we investigated upstream regulatory mechanisms, particularly the phosphorylation of p38 MAPK, a known activator of MEF2C [36]. Phosphorylated p38⁺NeuN⁺ cell numbers were markedly elevated in the EMMP-miKO group at both time points (Fig. 8i, j), suggesting enhanced MEF2C activity via p38 MAPK signaling.

Downstream of MEF2C, we examined the expression of anti-apoptotic Bcl2 in neurons as MEF2C has been reported to regulate Bcl2 expression [37]. EMMP-miKO mice showed significantly increased numbers of Bcl2⁺NeuN⁺ cells, along with a higher proportion of Bcl-2 signal within the ROI (Fig. 8k-m). Together, these findings support the involvement of a p38 MAPK/MEF2C/Bcl2 axis in mediating the neuroprotective effects observed upon EMMPRIN deletion.

### Targeted reduction of MEF2C by AAV abolishes the neuroprotection conferred by microglial EMMPRIN deletion after ICH

**Fig. 9:**
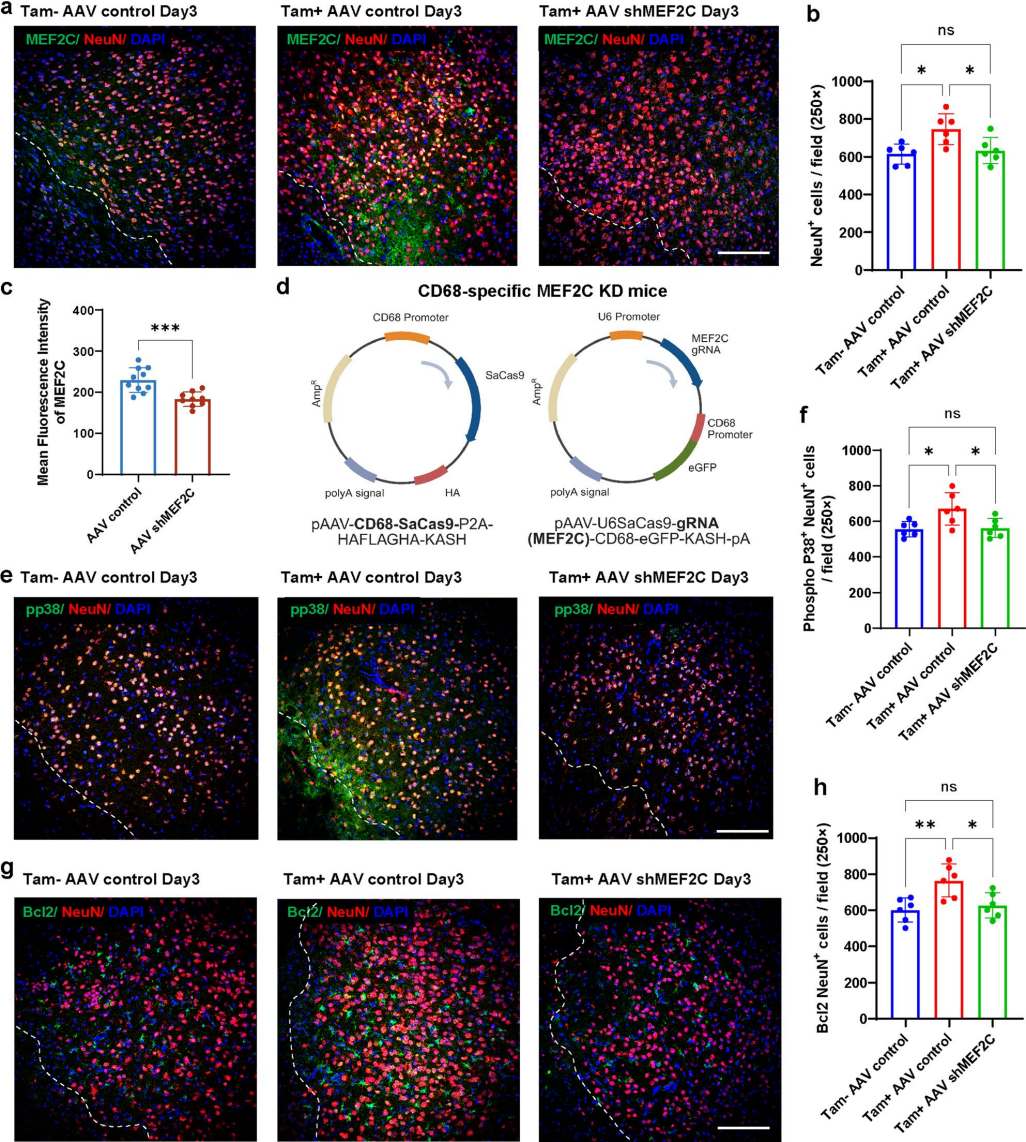
Targeted reduction of MEF2C by AAV abolishes the neuroprotection conferred by microglial EMMPRIN deletion after ICH. **(a)** Representative confocal images of MEF2C (green) co-expression with NeuN (red) in Tam − AAV control, Tam + AAV control and Tam + AAV-shMEF2C groups at day 3 post-ICH. The left corner within the dotted line of the perihematomal region indicates the center of the lesion. Scale bar, 100 μm. **(b)** Quantification of NeuN⁺ neurons per field (250×) in the three groups. **(c)** Mean fluorescence intensity of MEF2C in Tam + mice receiving AAV control or AAV-shMEF2C. **(d)** Schematic showing the genome of AAVs used in this study. AAVs were injected retro-orbitally at 3 × 10^11^ vg/virus per mouse 2 weeks before ICH for targeted reduction of MEF2C (KD) or non-target guide RNA (Ctrl). **(e)** Representative confocal images of phosphorylated p38 (pp38, green) co-localized with NeuN (red) in the indicated groups at day 3 post-ICH. Scale bar, 100 μm. **(f)** Quantification of pp38⁺NeuN⁺ cell density in the three groups. **(g)** Representative confocal images of Bcl2 (green) co-expression with NeuN (red) in the indicated groups at day 3 post-ICH. Scale bar, 100 μm. **(h)** Quantification of Bcl2⁺NeuN⁺ density in the three groups. Sample size in each group was *n* = 6. Data are presented as the mean ± SEM and analyzed by one-way ANOVA-Tukey’s post hoc test; ns: not significant. Significance is indicated as *p < 0.05, **p < 0.01, ***p < 0.001

To determine whether MEF2C is required for the neuroprotective effects associated with EMMPRIN deletion, we knocked down MEF2C using a dual AAV system in EMMP-miKO and control mice (Fig. 9). In tamoxifen-treated EMMP-miKO mice, AAV-shMEF2C markedly reduced MEF2C immunofluorescence in the perihematomal region compared with AAV control, confirming efficient knockdown (Fig. 9a, c).

Functionally, EMMP-miKO mice receiving AAV control retained the neuroprotective phenotype, with higher number of neurons at day 3 post-ICH compared with tamoxifen-untreated AAV control animals (Fig. 9a, b). This benefit was largely lost when MEF2C was knocked down: NeuN⁺ neuronal counts in Tam⁺ AAV-shMEF2C mice fell towards levels seen in Tam⁻ AAV controls (Fig. 9b).

Consistent with a role for the p38 MAPK/MEF2C/Bcl2 axis, the increase in pp38⁺NeuN⁺ neurons and Bcl2⁺NeuN⁺ neurons observed in Tam⁺ AAV control mice was blunted after MEF2C knockdown (Fig. 9e–h). For completeness, we also examined tamoxifen-untreated mice receiving AAV-shMEF2C and observed a further reduction in neuronal survival compared with the other three groups. Taken together, these findings indicate that MEF2C is necessary for the neuronal protection conferred by microglial EMMPRIN deletion after ICH.

### EMMPRIN deletion improves neurogenesis and oligodendrogenesis following ICH

**Fig. 10:**
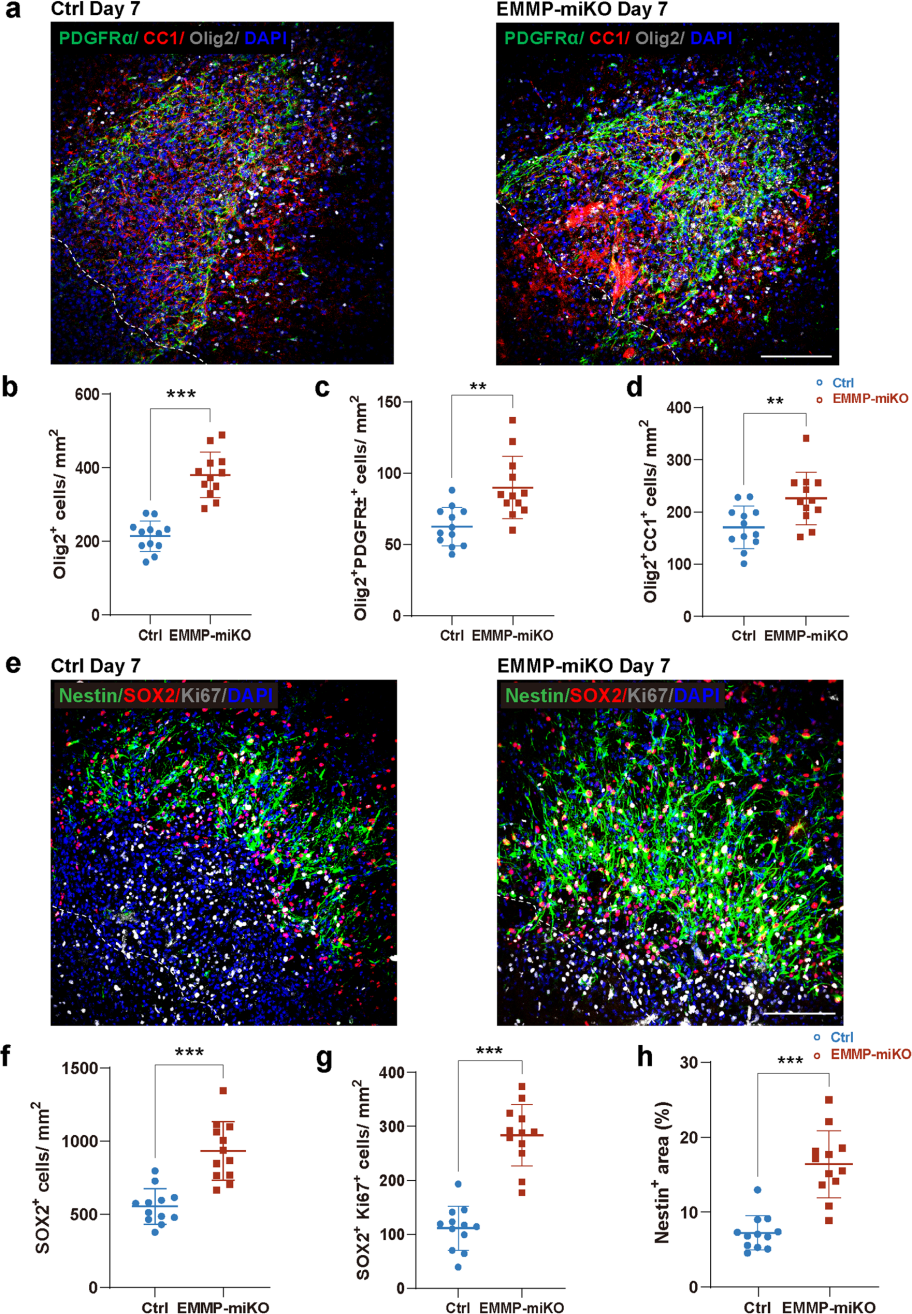
Microglial EMMPRIN knockout improves neurogenesis and oligodendrogenesis following ICH. **(a)** Representative confocal images of the perihematomal region at day 7 post-ICH in control (Ctrl) and EMMP-miKO mice. Pseudocolor labeling: PDGFRα (green), CC1 (red), Olig2 (gray), and DAPI (blue). The left corner within the dotted line of the perihematomal region indicates the center of the lesion. Scale bar, 100 μm. **(b–d)** Quantification of Olig2⁺ (b), Olig2⁺PDGFRα⁺ (c), and Olig2⁺CC1⁺ (d) cell densities in the perihematomal region of Ctrl and EMMP-miKO mice at day 7 post-ICH. **(e)** Representative confocal images of the perihematomal region at day 7 post-ICH, labeled for Nestin (green), SOX2 (red), Ki67 (gray), and DAPI (blue). The left corner within the dotted line of the perihematomal region indicates the center of the lesion. Scale bar, 100 μm. **(f–h)** Quantification of SOX2⁺ cell density (f), SOX2⁺Ki67⁺ cell density (g), and percentage area of Nestin⁺ regions (h) in Ctrl and EMMP-miKO groups at day 7 post-ICH. Sample size in the Ctrl group and the EMMP-miKO group was *n* = 15 on day 3 post-ICH; *n* = 12 on day 7 post-ICH. Data are presented as the mean ± SEM and analyzed by t-test; ns: not significant. Significance is indicated as **p < 0.01, ***P < 0.001

Given the reduced injury and neuronal death after ICH in the CX3CR1^Cre^:EMMPRIN^fl/fl^ mice, we investigated whether a later consequence would be neuroregeneration. We evaluated lesions at day 7 post-ICH and found that EMMP-miKO mice had elevated expression of oligodendrocyte lineage cells (Olig2^+^PDGFRα⁺ oligodendrocyte precursors and Olig2^+^CC1⁺ mature oligodendrocytes) in the perihematomal region compared to controls (Fig. 10a). Quantification showed a ~ 30% increase in Olig2⁺ (Fig. 10b, *p* < 0.001), a ~ 50% increase in Olig2⁺PDGFRα⁺ oligodendrocyte precursor cells (Fig. 10c, *p* < 0.01), and a significant rise in mature Olig2⁺CC1⁺ oligodendrocytes (Fig. 10d, *p* < 0.01). In parallel, EMMP-miKO mice exhibited increased neural stem cell representation, with denser Nestin⁺ networks and more SOX2⁺Ki67⁺ proliferating cells (Fig. 10e-h). Together, these findings highlight neuroreparative processes including neurogenesis and oligodendrogenesis occurring in EMMPRIN conditionally deleted mice after ICH.

## Discussion

Despite advances in supportive care, ICH remains a highly lethal and disabling form of stroke, largely due to the lack of effective interventions targeting secondary brain injury. A growing body of evidence implicates microglia/macrophages as central mediators of post-ICH neuroinflammation, extracellular matrix remodeling, and neuronal death. However, the molecular regulators orchestrating microglia/macrophage-driven pathology have not been fully elucidated. In this study, we identify EMMPRIN as a critical modulator of microglia/macrophage responses in ICH. Through genetic and AAV-mediated approaches, we demonstrate that targeted deletion of EMMPRIN mitigates neuronal death, reduces lesion volume, improves neurological function, and enhances regenerative responses. Mechanistically, our findings suggest that EMMPRIN exerts its deleterious effects via MMP-dependent extracellular matrix remodeling/degradation and may regulate neuroprotection through the p38 MAPK/MEF2C/Bcl2 signaling pathway.

Our results reveal that EMMPRIN is rapidly and selectively upregulated in Iba1⁺ microglia/macrophages within the perihematomal region during the acute phase of ICH. This spatial restriction aligns with the region of maximal blood-brain barrier disruption, inflammation, and neuronal death, supporting a functional role for EMMPRIN in the local amplification of injury. EMMPRIN is known to drive MMP expression in both physiological and pathological contexts [38, 39], and our findings extend these observations by showing that EMMPRIN co-localizes with MMP-9 and MMP-2 following ICH and that its suppression attenuates their expression. MMPs, particularly MMP-9 and MMP-2, are implicated in promoting vasogenic edema and neurotoxicity [40, 41], and are widely recognized as key mediators of blood-brain barrier disruption and secondary injury following ICH [24, 42]. Our findings position EMMPRIN as a key upstream regulator of the proteolytic cascade driving secondary brain injury.

To further delineate the functional role of EMMPRIN in microglia/macrophages, we employed two complementary loss-of-function strategies: an AAV-based CRISPR/Cas9 knockdown targeting CD68⁺ myeloid cells and a tamoxifen-inducible, microglia/macrophage-specific conditional knockout model (EMMP-miKO) driven by CX3CR1^CreER^. While both approaches effectively reduced EMMPRIN expression and conferred neuroprotection, they differed in timing, cellular specificity, and mechanistic implications. The AAV strategy enabled pre-ICH knockdown of EMMPRIN in a broader population of myeloid cells, including perivascular and infiltrating macrophages. This intervention suppressed MMP activation and improved early functional outcomes, accompanied by a decrease in pro-inflammatory IL-1β and a concomitant increase in arginase-1, a marker of reparative microglia/macrophage functional subtype [43]. These findings link myeloid cells to early inflammatory injury post-ICH and suggest that targeting EMMPRIN in these cells may modulate the initial immune microenvironment more broadly than previously appreciated. These observations are consistent with the concept of microglial functional plasticity [44, 45] and suggest that EMMPRIN may act as a molecular switch governing the balance between pro-inflammatory and regulatory or homeostatic microglial states. Previous studies have shown that targeting microglial activation alone can attenuate neuronal death and improve functional outcomes [46, 47]; however, our findings reveal that targeting upstream regulators like EMMPRIN within microglia/macrophages may offer broader benefits by modulating both inflammatory and matrix-degradative programs.

In contrast to AAV, the EMMP-miKO model provided selective and temporal ablation of EMMPRIN in microglia/macrophages. Despite the decline of EMMPRIN expression after day 3 of ICH in wildtype mice, microglia/macrophage-specific knockdown of EMMPRIN led to sustained reduction in Iba1⁺ density and significant improvement in neuronal survival and behavioral recovery. These results suggest that early microglial EMMPRIN signaling initiates downstream injury cascades with long-lasting consequences, paralleling prior studies implicating microglial priming in chronic neuroinflammation and secondary degeneration [48–50]. Together, these two models underscore the multifaceted role of EMMPRIN across distinct myeloid compartments and highlight microglia/macrophages as a central effector of its deleterious signaling. The convergence of outcomes across both strategies strengthens the causal link between EMMPRIN activity and ICH progression, while offering translational relevance for both prophylactic and cell-specific therapeutic targeting. A limitation of our study is that we did not discriminate EMMPRIN contribution by microglia versus macrophages, since the CX3CR1 promoter is in both myeloid populations. This will be a subject for future studies.

In addition to dampening injury-related processes, EMMPRIN deletion promoted regenerative responses, including neurogenesis and oligodendrogenesis. This is a notable finding, as the post-ICH brain has limited capacity for endogenous repair, and strategies that enhance remyelination and neurogenesis remain underdeveloped [51, 52]. The observed expansion of Olig2⁺, PDGFRα⁺, and CC1⁺ cells in EMMP-miKO mice indicates that microglial EMMPRIN may influence oligodendrocyte lineage dynamics, either directly through modulation of the extracellular environment, or indirectly by reducing the extent of injury in the first place. Moreover, the increase in SOX2⁺Ki67⁺ neural progenitors and Nestin⁺ regions suggests that microglial EMMPRIN also shapes the neurogenic niche. While the precise molecular link between microglial EMMPRIN and progenitor cell activation remains to be defined, our findings underscore the potential of EMMPRIN targeting not only to suppress injury but also to facilitate recovery.

Mechanistically, our proteomic analysis identified MEF2C as a downstream effector enriched in EMMPRIN-deficient brains. MEF2C is a transcription factor critical for neuronal survival, synaptic maintenance, and neurogenesis [53, 54]. MEF2C downregulation has been reported in multiple neurological disorders, including ischemia-reperfusion injury [55], Alzheimer’s disease [56], and spinal cord injury [57]. Consistent with these findings, we observed a marked reduction of MEF2C expression in ICH, which was reversed upon EMMPRIN deletion. Notably, MEF2C upregulation in NeuN⁺ neurons was accompanied by enhanced phosphorylation of p38 MAPK, a known upstream activator of MEF2C in neuroprotective signaling [58, 59]. Importantly, Bcl-2, an anti-apoptotic effector regulated by MEF2C [60], was also upregulated in neurons, supporting the existence of a p38 MAPK/MEF2C signaling pathway. AAV-based knockdown of MEF2C further strengthens this model, as suppression of MEF2C largely abolished the gain in NeuN⁺ neuronal survival, so that neuronal density returned to that of control animals. These loss-of-function data indicate that MEF2C is required for the neuroprotective effect of microglial EMMPRIN deletion rather than acting as a passive marker of reduced injury. Beyond this downstream axis, our proteomic data point to upstream regulation involving extracellular-matrix and inflammatory pathways, with enrichment of complement and vascular–inflammatory signaling and representative changes in ECM and acute-phase components (e.g. ITIH3, Serpina1e, angiotensinogen, Gnas and Syngap1). We therefore propose that EMMPRIN-dependent remodeling of the perihematomal microenvironment engages upstream receptor systems and ultimately drives neuronal p38/MEF2C/Bcl-2 activation. This hypothesis provides a framework for future studies aimed at defining the precise ligand–receptor and signaling modules involved.

Our study has several important implications. First, it highlights EMMPRIN as a critical mediator of microglia/macrophage-driven pathology in ICH. By promoting MMP activation and inflammatory polarization, EMMPRIN contributes to BBB disruption, neuronal death, and impaired recovery. Its deletion not only attenuates these detrimental processes but also facilitates neuroregeneration. Second, by demonstrating that EMMPRIN deletion enhances regenerative processes, this study suggests that intervention may not only limit damage but also support recovery, a dual benefit that is rarely achieved in stroke therapy. Finally, the identification and functional validation of MEF2C as a downstream effector target opens opportunities to explore transcriptional reprogramming as a therapeutic strategy in ICH and other neuroinflammatory conditions.

## Conclusions

In conclusion, our findings identify EMMPRIN as a central mediator of microglia/macrophage-driven neurotoxicity and impaired repair following ICH. Targeted deletion of EMMPRIN in microglia/macrophages attenuates neuroinflammation, reduces neuronal death, and improves functional recovery, providing strong preclinical support for its therapeutic potential. Targeting EMMPRIN offers a promising strategy to mitigate secondary brain injury and enhance neuroregeneration in hemorrhagic stroke.

## Supplementary Information

Below is the link to the electronic supplementary material.

Supplementary Material 1

Supplementary Material 2

Supplementary Material 3

## Data Availability

Data and reagents are available from VWY and MX upon reasonable request.

## Abbreviations

AAV: Adeno-associated virus
BBB: Blood-brain barrier
BSA: Bovine serum albumin
DAPI: 4′,6-diamidino-2-phenylindole
EC: Eriochrome cyanine
EDTA: Ethylenediaminetetraacetic acid
EMMPRIN/CD147: Extracellular matrix metalloproteinase inducer
FASP: Filter-assisted sample preparation
GO: Gene Ontology
HCD: Higher-energy collisional dissociation
ICH: Intracerebral hemorrhage
IL-1β: Interleukin-1 beta
MAPK: Mitogen-activated protein kinase
MEF2C: Myocyte enhancer factor 2 C
MMP: Matrix metalloproteinase
MS: Mass spectrometry
NaCl: Sodium chloride
PFA: Paraformaldehyde
PPI: Protein–protein interaction
PRIDE: Proteomics Identifications Database
qPCR: Quantitative polymerase chain reaction
ROI: Region of interest
RPM: Revolutions per minute
SAH: Subarachnoid hemorrhage
SEM: Standard error of the mean
SDS: Sodium dodecyl sulfate
sgRNA: Single-guide RNA
tMCAO: Transient middle cerebral artery occlusion
