# Supplementary material for: Targeted deletion of EMMPRIN in microglia/macrophages mitigates neuronal death in intracerebral hemorrhage
Source: Mol Neurodegener. 2025 Dec 13;21:4. doi: 10.1186/s13024-025-00917-x (PMC12817446; doi:10.1186/s13024-025-00917-x)
Supplement: Supplementary file 2 — Supplementary Material 2 [file 13024_2025_917_MOESM2_ESM.docx]

| **Target** | **Fluorophore** | **Clone** | **Company** | **Cat no.** | **Dilution** |
| --- | --- | --- | --- | --- | --- |
| CD45 | BUV395 | 30-F11 | BD Biosciences | 564279 | 1:200 |
| CD11b | BUV805 | M1/70 | BD Biosciences | 568345 | 1:200 |
| Ly6G | PerCP | 1A8 | Biolegend | 127654 | 1:100 |
| Ly6C | BV570 | HK1.4 | Biolegend | 128030 | 1:200 |
| CD11c | BV711 | N418 | Biolegend | 117349 | 1:100 |
| CD3 | APC-Fire810 | 17A2 | Biolegend | 100268 | 1:100 |
| F4/80 | PE-Cy7 | BM8 | Biolegend | 123114 | 1:100 |
| B220 | BUV737 | RA3-6B2 | BD Biosciences | 612839 | 1:300 |
| MHC-II | BV480 | M5/114.15.2 | BD Biosciences | 566086 | 1:200 |
| CD86 | AF488 | GL-1 | Biolegend | 105018 | 1:200 |
| CD206 | BV650 | C068C2 | Biolegend | 141723 | 1:100 |

**Supplementary Table S1.** Antibodies used for flow cytometry.

**Supplemental Table S2.** Primary and secondary antibodies used in the study

| **Target** | **Antibody** | **Dilution** | **Company** | **Cat no.** |
| --- | --- | --- | --- | --- |
| EMMPRIN | Rat anti-mouse EMMPRIN | 1:100 | Biolegend | OX-114 |
| Microglia/  macrophages | Rabbit anti-human/mouse Iba1 | 1:1000 | Wako | 019-19741 |
| Microglia/  macrophages | Goat anti-human/mouse Iba1 | 1:250 | ThermoFisher | PA5-18039 |
| Astrocytes | Chicken anti-mouse GFAP | 1:1000 | Biolegend | 829401 |
| Neuron | Rabbit anti-human/mouse NeuN | 1:500 | Abcam | ab177487 |
| Endothelial cell | Rat anti-mouse CD31 | 1:200 | BD Pharmingen | 550274 |
| Microglia/  macrophages | Rat anti-mouse CD68 | 1:500 | Biolegend | 137002 |
| Neutrophils | Rat anti-mouse Ly6G | 1:100 | Biolegend | 127606 |
| Matrix metalloproteinase 9 | Rabbit anti-mouse MMP9 | 1:500 | Abcam | ab76003 |
| Matrix metalloproteinase 2 | Rabbit anti-mouse MMP2 | 1:200 | Proteintech | 10373-2-AP |
| Arg 1 | Rabbit anti-mouse  arginase 1 | 1:200 | Cell Signaling | 93668S |
| IL-1β | Goat anti-mouse interleukin-1β | 1:50 | R&D Systems | AF-401-NA |
| PDGFRα | Goat anti-mouse platelet-derived growth factor receptor | 1:200 | R&D Systems | AF1062 |
| CC1 | Mouse anti-human/mouse adenomatous polyposis coli (APC) | 1:200 | Millipore | OP80-100UG |
| Oligodendrocyte | Rabbit anti- human/mouse Olig2 | 1:200 | Millipore | AB9610 |
| Nestin | Chicken anti-human/mouse nestin | 1:1000 | Novusbio | NB100-1604 |
| SOX2 | Rat anti-human/ mouse SOX2 | 1:200 | ThermoFisher | 14-9811-82 |
| Ki67 | Rabbit anti-human/mouse Ki67 | 1:500 | Abcam | AB15580 |
| MEF2C | Mouse anti-human/mouse MEF2C | 1:100 | Novus | OTI1H5 |
| Phospho-p38 | Rabbit anti-mouse phospho-p38 | 1:800 | Cell Signaling | 4511 |
| Bcl-2 | Goat anti-human/mouse Bcl-2 | 1:20 | R&D Systems | AF810 |
| ZO1 | Rabbit anti-rat/human  ZO1 | 1:1000 | Abcam | AB221547 |
| Mouse IgM | Alexa Fluor 488 donkey anti-mouse IgM | 1:400 | ImmunoResearch Jackson | 715-545-140 |
| Goat IgG | Alexa Fluor 488 donkey anti-goat IgG | 1:400 | ImmunoResearch Jackson | 705-545-147 |
| Rabbit IgG | Alexa Fluor 647 donkey anti-rabbit IgG | 1:400 | ImmunoResearch Jackson | 711-605-152 |
| Chicken IgY | Cyanine Cy3 donkey anti-chicken IgY | 1:400 | ImmunoResearch  Jackson | 703-165-155 |
| Goat IgG | Cyanine Cy3 donkey anti-goat IgG | 1:400 | ImmunoResearch Jackson | 705-165-147 |
