# Supplementary material for: Targeted deletion of EMMPRIN in microglia/macrophages mitigates neuronal death in intracerebral hemorrhage
Source: Mol Neurodegener. 2025 Dec 13;21:4. doi: 10.1186/s13024-025-00917-x (PMC12817446; doi:10.1186/s13024-025-00917-x)
Supplement: Supplementary file 3 — Supplementary Material 3 [file 13024_2025_917_MOESM3_ESM.docx]

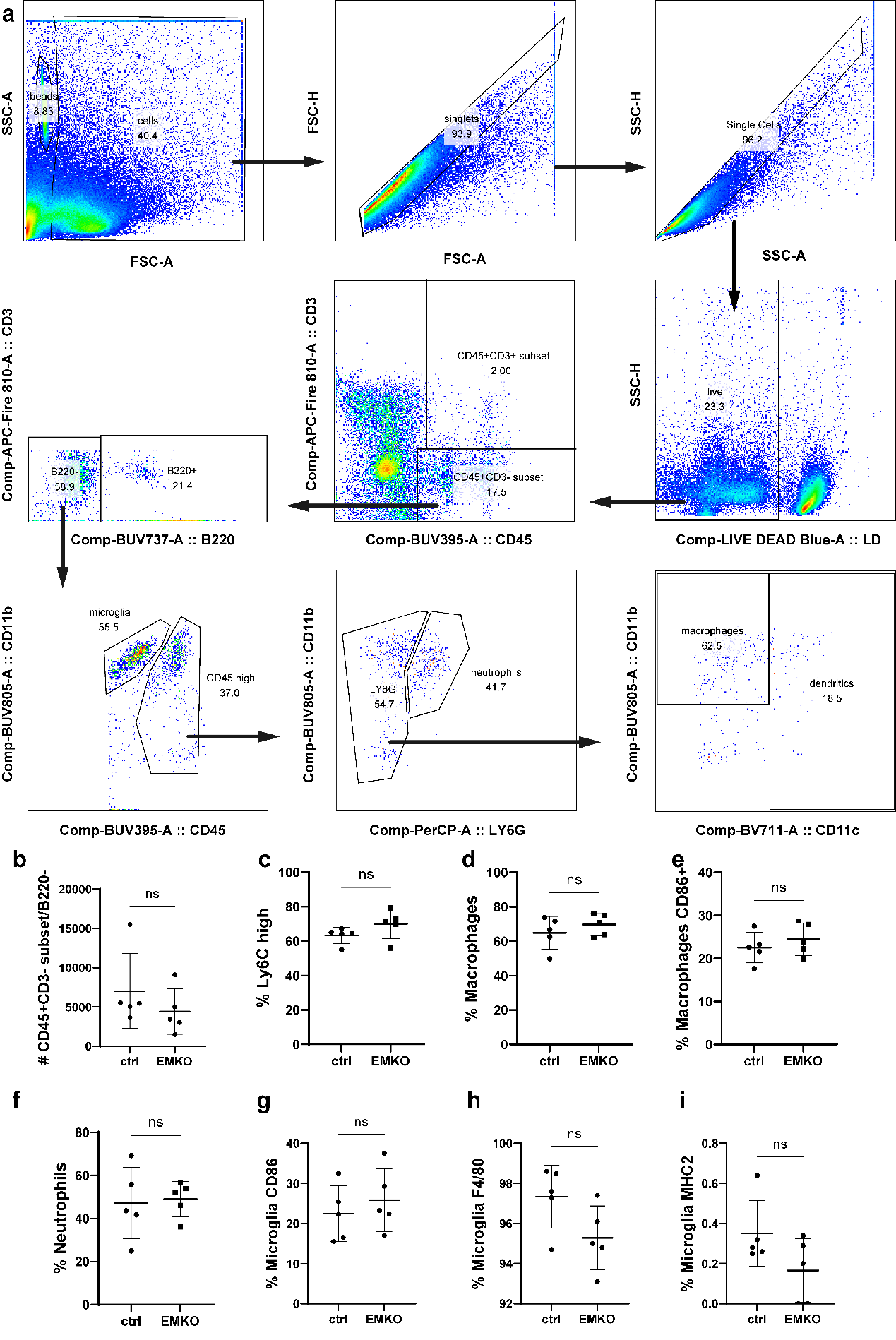


**Supplementary Figure S1. Gating strategy for brain immune cells after ICH. (a)** Events were first cleaned by sequential gates on forward and side scatter to exclude debris and counting beads (FSC-A vs SSC-A), followed by singlet gates (FSC-H vs FSC-A and SSC-H vs SSC-A) and a live gate based on the LIVE/DEAD Blue viability dye. Leukocytes were then identified as CD45⁺ events. Within the CD45⁺ population, lymphoid and myeloid compartments were resolved according to CD3, B220 and CD11b expression. T cells were defined as CD45⁺CD3⁺ cells, whereas B cells were defined as CD45⁺CD3⁻B220⁺ cells. Non-lymphoid cells were analysed in the CD45⁺CD3⁻B220⁻ gate. Microglia were identified as CD45intCD11b⁺ cells, and infiltrating myeloid cells as CD45hiCD11b⁺ cells. Within the CD45hiCD11b⁺ fraction, neutrophils were gated as Ly6G⁺ cells, while Ly6G⁻ cells were further separated on CD11c expression, with CD11c⁺ cells classified as dendritic cells and CD11c⁻ cells as macrophages. **(b–i)** Quantification of myeloid and microglial subsets and activation markers in ctrl and EMKO mice: **(b)** number of CD45⁺CD3⁻B220⁻ myeloid cells; **(c–f)** proportions of Ly6G⁺ neutrophils and CD11b⁺CD11c⁻ macrophages and CD86⁺ macrophages among the CD45⁺CD3⁻B220⁻ compartment; **(g–i)** percentages of CD86⁺, F4/80⁺ and MHC II⁺ cells among CD45^int^CD11b⁺ microglia. Sample size in both group was n=5; Data are presented as mean ± SEM; ns, not significant.
